# SHAP-Based Feature Augmentation and Stacking Ensemble Learning for ECG-Based Serum Potassium Abnormality Prediction

**DOI:** 10.3390/bioengineering13070816

**Published:** 2026-07-16

**Authors:** Yi-Hsin Ko, Chuan-Sheng Hung, Chun-Hung Richard Lin, Yi-Fong Ciou, Chiang-Chi Huang, Pin-Chen Lin, Jui-Hsiu Tsai

**Affiliations:** 1 Department of Computer Science and Engineering, National Sun Yat-sen University, Kaohsiung 804, Taiwan; zxc78945606nsysu@gmail.com (Y.-H.K.); lin@cse.nsysu.edu.tw (C.-H.R.L.); m133040023@student.nsysu.edu.tw (Y.-F.C.); herme381981@gmail.com (C.-C.H.); m123040010@student.nsysu.edu.tw (P.-C.L.); 2 Autonomous UAV & AI Robotics Research Center, National Sun Yat-sen University, Kaohsiung 804, Taiwan; 3 Artificial Intelligence Research and Promotion Center, National Sun Yat-sen University, Kaohsiung 804, Taiwan; 4 Center of Teaching and Research, Kaohsiung Municipal Siaogang Hospital, Kaohsiung 812, Taiwan; 5 Division of Nephrology, Department of Internal Medicine, Kaohsiung Chang Gung Memorial Hospital and Chang Gung University College of Medicine, Kaohsiung 833, Taiwan; 6 School of Medicine, Tzu Chi University, Hualien 970, Taiwan; 7 Department of Psychiatry, Dalin Tzu Chi Hospital, Buddhist Tzu Chi Medical Foundation, Chiayi 622, Taiwan

**Keywords:** feature augmentation, explainable artificial intelligence, electrocardiogram, machine learning, time-series classification

## Abstract

Electrocardiogram (ECG) signals contain important clinical information associated with serum potassium abnormalities. However, in Taiwan, raw patient data and original medical signals generally cannot be taken outside the hospital environment, thereby limiting their subsequent reuse and cross-institutional applications. The objective of this study is to transform classification-related information contained in raw ECG signals into high-level SHAP features and to evaluate their feasibility as auxiliary or alternative features to the original wave-segment model outputs. To this end, this study proposes a time-series classification framework that integrates ECG wave-segment submodels, SHAP-based feature augmentation, and stacking ensemble learning for serum potassium abnormality prediction. Submodels are first trained separately using different ECG wave segments and their combinations. SHAP is then applied to transform the contributions of the wave-segment submodel outputs to the prediction results into high-level features. In addition, PCA features are included as a comparison baseline to analyze the effects of different feature transformation methods on classification performance. The experimental results show that incorporating SHAP-based augmented features improves ECG-based serum potassium abnormality prediction performance under most settings. Even when only SHAP-based augmented features are used for training, some models still maintain performance comparable to or better than the Baseline. Although PCA provides more stable classification balance for some patients, SHAP-based augmented features can still represent classification-related model contribution information under most settings while achieving better or comparable classification performance. Overall, even without directly using the original ECG signals in the final classification stage, these features retain a certain degree of discriminative information and demonstrate the potential to serve as alternative features to the original wave-segment model outputs. Therefore, the findings of this study provide a preliminary reference for future medical data reuse, cross-institutional collaboration, and privacy risk assessment.

## 1. Introduction

In recent years, artificial intelligence (AI) and machine learning techniques have been widely applied to medical data analysis, disease classification, diagnosis, disease prediction, and prognostic assessment [1,2,3]. Among these applications, time series classification (TSC) is of substantial value in medical signal analysis because it can identify specific patterns from data with temporal dependencies. Existing studies have indicated that various TSC methods have been applied to biomedical data analysis, industrial inspection, and multivariate time-series anomaly detection, and have achieved considerable classification performance in biomedical applications [1,4]. Electrocardiography (ECG), as a representative type of physiological time-series data, reflects cardiac electrophysiological activity and has been used to detect electrolyte imbalance conditions such as serum potassium abnormalities. Hyperkalemia is a clinically important electrolyte disorder commonly observed in patients with chronic kidney disease and may also occur in patients with diabetes mellitus or heart failure. If not detected and managed in a timely manner, it may increase the risk of adverse clinical outcomes [5]. Previous studies have shown that serum potassium abnormalities may induce severe arrhythmias, whereas conventional serum potassium testing requires blood sampling and is therefore less suitable for frequent monitoring. In contrast, ECG is noninvasive, rapid, and repeatable, making it an important signal source for assisting the detection of serum potassium abnormalities [6].

Over the past decade, data science research has continuously sought to develop AI-based models for analyzing ECG waveform characteristics to accurately identify and classify abnormal cardiac rhythms [2]. However, in high-risk medical settings, models that lack sufficient transparency and interpretability may hinder clinicians from understanding their decision logic and establishing trust [7,8]. Related studies have also indicated that model explainability in biomedical time-series classification can help interpret model decisions and improve the clinical value of predictive models [9]. In addition to the need for interpretability, the practical deployment of medical AI also faces challenges related to clinical data sharing. Because medical data are highly sensitive, privacy protection must be maintained throughout the entire process from model training to inference. Moreover, cross-institutional data sharing in healthcare is often challenging because of complex and varying privacy and regulatory requirements [10,11]. Therefore, if the construction and validation of medical AI models rely heavily on raw clinical data, cross-hospital data acquisition and external validation may still be affected by institutional approval procedures and data governance requirements. This limitation may prevent the model from sufficiently learning population differences across regions and medical institutions, thereby reducing its generalizability and practical value in multicenter clinical settings.

At the feature representation level, although raw ECG signals preserve complete waveform information, different waveform segments may carry distinct physiological meanings and may contribute differently to the prediction outcome. For example, a recent study on ECG-based dyskalemia detection further compared the importance of the P, QRS, and T waves for model prediction. The results showed that the P wave had a relatively limited impact on model performance, whereas masking the QRS complex and T wave led to a marked decrease in the area under the receiver operating characteristic curve (AUROC), a metric used to evaluate the overall discriminative ability of a classification model across different thresholds, indicating that different ECG wave segments possess varying degrees of discriminative value for predicting serum potassium abnormalities [6].

Since the objective of this study is to detect serum potassium abnormalities from ECG signals, directly feeding the complete continuous ECG signal into the model may cause the model to be simultaneously affected by differences in heartbeat cycle length, local waveform variations, and overall signal fluctuations. Therefore, this study first segments the raw ECG signal into individual heartbeat segments, and each heartbeat cycle is regarded as a temporal subsequence with a representative waveform structure, namely a shapelet in time-series classification. Through this processing strategy, the model can use each individual heartbeat as the basic analytical unit and learn local waveform features within each heartbeat cycle that are associated with serum potassium status.

However, if learning relies only on raw ECG signals or basic waveform features, the model may still extract information from the overall signal, but it may not explicitly reveal the relative contribution of each wave segment to the prediction outcome, nor can it easily highlight the key feature patterns that are truly useful for identifying serum potassium abnormalities. In recent years, explainable artificial intelligence (XAI) methods have also been increasingly used to improve model prediction performance. Related studies have begun to examine whether the interpretable information revealed by models can be further transformed into reusable feature representations to enhance model performance and learning quality. Among these methods, Shapley-based feature augmentation has shown through experimental results that incorporating Shapley values generated by XAI methods as augmented features can provide models with additional feature contribution information, enabling classification models to better exploit discriminative signal patterns and improve predictive performance [12].

To this end, this study uses ECG data as the research target and proposes a time-series classification framework incorporating SHAP (SHapley Additive exPlanations) [13] feature augmentation for the task of serum potassium abnormality detection. First, since different wave segments reflect different cardiac electrophysiological activities and may contain varying degrees of discriminative information for serum potassium abnormality detection, the ECG signals are segmented according to heartbeat waveforms into P, QRS, T, and their combined wave segments. Classification models are then trained separately for each segment to capture local signal morphology within individual wave segments as well as the overall dynamic characteristics represented by different wave-segment combinations. Next, the prediction outputs of the wave-segment models are used as the basis for generating SHAP features, thereby quantifying the contribution of different wave-segment outputs to the final prediction outcome and transforming this contribution information into additional features for feature augmentation. Finally, stacking is used to integrate the outputs of the wave-segment models and the augmented feature information, thereby improving overall classification stability and generalization ability. To further validate the practical effectiveness of SHAP features, this study designs three input settings for comparison: first, the Baseline setting, which uses only the original prediction outputs of the wave-segment models; second, the SHAP+Original feature setting, which combines the original wave-segment outputs with SHAP-augmented features; and third, the SHAP-only feature setting, which uses only the contribution information transformed by SHAP as the model input. Through this design, this study compares the effects of SHAP features as auxiliary information and as alternative feature representations on the final classification performance and stability. Meanwhile, this study also incorporates PCA (Principal Component Analysis) [14] feature augmentation as a comparative benchmark to evaluate the practical effectiveness of different feature representation methods in ECG-based serum potassium abnormality detection.

The contributions of this study are threefold. First, we propose an ECG time-series classification framework that integrates seven ECG wave segments with stacking ensemble learning. Through the submodel outputs of the P, QRS, T, and their combined wave segments, the proposed framework integrates the local morphology and overall dynamic information embedded in different ECG regions. Second, this study further transforms SHAP from a conventional model interpretation tool into reusable high-level augmented features. By using the outputs of the wave-segment submodels as the targets of SHAP analysis, the relative contributions of different ECG wave segments to serum potassium abnormality detection are quantified, thereby validating their discriminative ability and interpretive value. Third, by comparing different input settings, including Baseline, SHAP+Original features, SHAP-only features, and PCA-based feature augmentation, this study verifies the effectiveness of SHAP features as both auxiliary information and alternative feature representations. The experimental results show that SHAP features not only enhance classification performance but also exhibit feasibility when used independently as model inputs, providing a preliminary application basis for future studies on feature-level cross-hospital model sharing and privacy-preserving learning.

## 2. Related Works

In recent years, artificial intelligence has been increasingly applied to medical signal analysis and clinical decision support. ECG has also been progressively used for arrhythmia recognition, disease risk prediction, and other clinical interpretation tasks [15]. In studies related to hyperkalemia, previous clinical evidence has reported that patients with hyperkalemia may present specific ECG abnormalities, suggesting an important association between ECG manifestations and serum potassium abnormalities [16]. Furthermore, existing studies have demonstrated that deep learning models can identify hyperkalemia and hypokalemia from ECG signals and maintain favorable performance on external validation data [6]. Accordingly, the following section reviews and discusses the development of ECG-based serum potassium abnormality detection, model construction strategies, and clinical applications.

An et al. [6] used clinical data from two hospitals between 2006 and 2020 to establish paired datasets of ECG recordings and serum potassium test results, and developed deep learning models to detect hyperkalemia ($K^{+}\geq 5.5\;\mathrm{mEq}/L$) and hypokalemia ($K^{+}<3.5\;\mathrm{mEq}/L$). In this study, 12-lead ECG, limb-lead ECG, and lead I ECG were used as model inputs, and end-to-end analysis was performed directly on raw ECG signals to evaluate the feasibility of using ECG for dyskalemia screening under different numbers of leads. The results showed that the 12-lead ECG model achieved AUROCs of 0.929 and 0.925 for detecting hyperkalemia and hypokalemia, respectively, in the internal test dataset, and also achieved AUROCs of 0.923 and 0.913 in the external validation dataset. These findings indicate the potential of ECG as a noninvasive signal source for detecting serum potassium abnormalities. The study further interpreted the model by masking the P wave, QRS complex, and T wave, and found that the P wave had a relatively limited impact on model performance, whereas masking the QRS complex and T wave led to a marked decrease in AUROC. This suggests that different ECG wave segments have varying degrees of discriminative value for predicting serum potassium abnormalities.

Galloway et al. [17] proposed a deep learning model for screening hyperkalemia from ECG signals. This study used large-scale clinical data collected by the Mayo Clinic between 1994 and 2017 for model development. The model development dataset included 449,380 patients and 1,576,581 ECG recordings, with serum potassium test results obtained within 12 h before or after the ECG used as the labeling basis. A serum potassium level of 5.5 mEq/L or higher was defined as hyperkalemia. The study population mainly focused on patients with stage 3 or more advanced chronic kidney disease (CKD), as these patients are at higher risk of hyperkalemia and often require clinical potassium monitoring. The authors trained a deep convolutional neural network using either two ECG leads (leads I and II) or four ECG leads (leads I, II, V3, and V5) as inputs. After receiving a 10 s ECG signal, the model generated a prediction score between 0 and 1 as the basis for noninvasive hyperkalemia screening. In the validation results, when two ECG leads were used, the model achieved AUCs of 0.883, 0.860, and 0.853 in the Minnesota, Florida, and Arizona validation datasets, respectively. At the high-sensitivity operating point, the sensitivity across the three validation datasets ranged from 88.9% to 91.3%, and the negative predictive value ranged from 99.0% to 99.6%, indicating that the model is more suitable as a noninvasive screening tool for ruling out hyperkalemia. These results demonstrate that deep learning models can capture ECG changes associated with hyperkalemia and have the potential to serve as clinical noninvasive screening tools.

Chiu et al. [18] proposed a personalized hyperkalemia detection method based on deep transfer learning. This study used the publicly available MIMIC-III Waveform Database Matched Subset and included intensive care unit patients with serum potassium test results and corresponding ECG signals. Single-lead ECG recordings obtained within 10 min before serum potassium testing were used for analysis. Methodologically, the study first trained a general 1D ResNet deep learning model using ECG heartbeat segments from a large number of patients. Then, for individual patients with multiple hyperkalemic and normokalemic records, personal ECG data were used for transfer learning and fine-tuning, enabling the model to adapt to inter-patient differences in ECG morphology. The experimental results showed that the personalized transfer learning model substantially improved hyperkalemia detection performance. Accuracy increased from an average of 0.604 for the general model to 0.980 for the personalized model, while AUC increased from 0.729 to 0.945. These findings indicate that, after incorporating individual patient data for fine-tuning, the model can more effectively adapt to ECG morphological differences across patients and thereby improve hyperkalemia recognition performance. The study concluded that single-lead ECG combined with personalized transfer learning has the potential to serve as a tool for continuous hyperkalemia monitoring and early warning.

Studies on ECG-based serum potassium abnormality detection have demonstrated, from multiple perspectives, the association between ECG signals and dyskalemic states. Related studies have not only examined the effects of different numbers of ECG leads and signal input formats on model performance but have also further analyzed the importance of different wave segments, such as the P wave, QRS complex, and T wave, to prediction outcomes. In addition, personalized model adaptation has been explored to reduce the recognition difficulties caused by inter-patient ECG variability. Overall, existing studies have shown that discriminative feature information can be extracted from ECG waveforms through model construction and signal analysis. However, a review of the relevant literature indicates that most studies still focus primarily on model architecture design, improvement of classification performance, clinical screening applications, or analysis of interpretability results, with relatively limited attention given to how model-generated explanatory information can be transformed into reusable feature representations. Therefore, this study further uses SHAP as a source of feature augmentation to investigate the feasibility and effectiveness of transforming explanatory information into auxiliary feature representations for ECG-based serum potassium abnormality detection.

## 3. Proposed Analysis Strategy and Model Architecture

This study aims to establish a time-series classification framework based on SHAP feature augmentation, using ECG-based serum potassium abnormality detection as the application case. Since ECG is a physiological signal that varies over time, this study treats each individual heartbeat as a fixed-length univariate time-series sample and uses serum potassium status as the binary classification target. Overall, the proposed framework consists of five main modules: ECG heartbeat segmentation, seven-wave-segment data construction, wave-segment submodel learning, SHAP feature augmentation, and stacking ensemble classification.

Let the dataset of the *s*-th patient be denoted as follows:

(1) $$D^{\left(s\right)}={\left\{\left(X_{i}^{\left(s\right)},y_{i}^{\left(s\right)}\right)\right\}}_{i=1}^{N_{s}}$$

where $X_{i}^{\left(s\right)}$ denotes the *i*-th ECG heartbeat time-series sample of the *s*-th patient, and $y_{i}^{\left(s\right)}\in \left\{0,1\right\}$ denotes its corresponding serum potassium status label, where 0 represents normokalemia and 1 represents serum potassium abnormality. In addition, $N_{s}$ denotes the number of samples for the *s*-th patient. Considering that ECG morphology and serum potassium variation may differ across patients, this study adopts a personalized modeling strategy, in which a separate classification model is constructed for each patient.

To analyze the effects of different ECG waveform segments on serum potassium abnormality detection, each heartbeat sample is transformed into seven wave-segment representations according to the ECG heartbeat structure. The set is defined as follows:

(2) $$B=\left\{P,\mathrm{QRS},T,P+\mathrm{QRS},\mathrm{QRS}+T,P+T,P+\mathrm{QRS}+T\right\}$$

where P, QRS, and T represent ECG waveform segments with distinct physiological meanings, whereas P+QRS, QRS+T, P+T, and P+QRS+T denote different combinations of wave segments. Through this design, the original ECG heartbeat can not only be analyzed as a complete time series but also be further decomposed into multiple physiologically meaningful wave-segment representations, allowing the effects of different wave segments on the serum potassium abnormality classification task to be examined.

In terms of model architecture, this study constructs time-series submodels separately for the seven wave-segment datasets of each patient, and the submodels corresponding to each wave segment generate preliminary prediction results. The outputs of the seven wave-segment models are then integrated as the input representation for the stacking stage, enabling the final classifier to simultaneously utilize the decision information provided by different ECG wave segments. Furthermore, this study incorporates the SHAP method into the proposed framework to transform model interpretation information into reusable augmented features. As a result, the final classifier can use not only the original outputs of the wave-segment models but also the contribution information of each wave segment to the prediction outcome.

In summary, the proposed framework is built on personalized ECG heartbeat time-series data. It first captures information from different ECG waveform segments through seven wave-segment representations, then integrates the decision outputs of multiple wave segments using wave-segment submodels and a stacking ensemble strategy. Finally, SHAP is further employed to transform model interpretation information into reusable augmented features. This framework is mainly designed to verify the overall utility of different ECG wave segments, wave-segment model outputs, and SHAP-augmented features in the task of serum potassium abnormality detection.

## 4. Method

**Figure 1:**
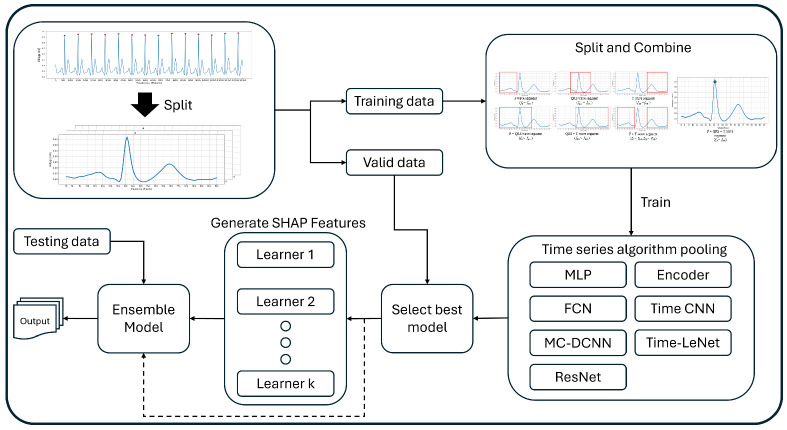
Study framework.

The workflow of this study is shown in Figure 1. The research objective is to determine the serum potassium status corresponding to ECG signals, where normokalemia is defined as y=0, and serum potassium abnormality is defined as y=1. The overall framework takes ECG signals as the analysis target and sequentially consists of heartbeat segmentation and wave-segment processing, seven-wave-segment submodel training, SHAP feature augmentation, and final stacking ensemble classification. Through this workflow, the raw ECG signals are progressively transformed into wave-segment-level model outputs and SHAP-augmented features. The decision information provided by different wave segments and feature representations is then further integrated to complete serum potassium status prediction.

### 4.1. Heartbeat Segmentation and Wave-Segment Processing

**Figure 2:**
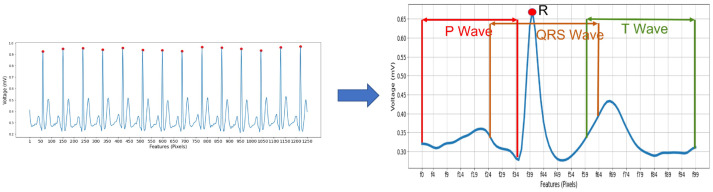
Schematic illustration of wave-segment division and combination of ECG heartbeat segments.

The heartbeat segmentation and wave-segment division procedure in this study consists of two levels. First, the original continuous ECG signal is segmented into individual heartbeat samples of fixed length 100 based on the R peak, so that each sample has a consistent time-series length. Second, according to the physiological structure of the ECG waveform, each heartbeat segment is further transformed into seven wave-segment datasets, including P, QRS, T, P+QRS, QRS+T, P+T, and P+QRS+T, as illustrated in Figure 2. This design enables the subsequent models to separately learn the local waveform features of individual wave segments and the overall dynamic information formed by different wave-segment combinations, thereby serving as the basis for subsequent wave-segment submodel training and ensemble classification.

Let the raw ECG signal of the *s*-th patient be denoted as follows:

(3) $$S^{\left(s\right)}={\left[s_{t}^{\left(s\right)}\right]}_{t=1}^{T_{s}}=\left[s_{1}^{\left(s\right)},s_{2}^{\left(s\right)},\ldots ,s_{T_{s}}^{\left(s\right)}\right]$$

Here, $S^{\left(s\right)}$ denotes the raw ECG signal sequence of the *s*-th patient, $s_{t}^{\left(s\right)}$ denotes the ECG signal value of the *s*-th patient at the *t*-th sampling point, $t=1,2,\ldots ,T_{s}$, and $T_{s}$ represents the total sampling length of the raw ECG signal for the *s*-th patient. To transform the continuous ECG signal into fixed-length heartbeat-level samples, this study uses the R peak as the reference point for heartbeat segmentation. The R peak is the major peak within the QRS complex, and its location can be used to identify an individual heartbeat cycle. In this study, R peaks are detected using the adaptive R-peak detection algorithm proposed by Christov [19], implemented in Python 3.10.18.

(4) $$R^{\left(s\right)}=\left\{r_{1}^{\left(s\right)},r_{2}^{\left(s\right)},\ldots ,r_{N_{s}}^{\left(s\right)}\right\}$$

Here, $r_{i}^{\left(s\right)}$ denotes the position of the *i*-th detected R peak for the *s*-th patient, and $N_{s}$ denotes the number of available heartbeat segments for that patient. For each R-peak position $r_{i}^{\left(s\right)}$, this study performs fixed-length segmentation centered on that point by extracting 40 sampling points to the left and 60 sampling points to the right, forming an individual heartbeat segment with a length of 100. The *i*-th heartbeat segment can be defined as follows:

(5) $$X_{i}^{\left(s\right)}=\left[s_{r_{i}^{\left(s\right)}-40}^{\left(s\right)},s_{r_{i}^{\left(s\right)}-39}^{\left(s\right)},\ldots ,s_{r_{i}^{\left(s\right)}+59}^{\left(s\right)}\right]\in R^{100}$$

$X_{i}^{\left(s\right)}$ denotes the *i*-th individual heartbeat time-series sample of the *s*-th patient. Here, $R^{100}$ indicates that each heartbeat segment is a one-dimensional real-valued vector of length 100. Since the sampling frequency of the data used in this study is 125Hz, each heartbeat segment with a length of 100 corresponds to approximately $\frac{100}{125}=0.8$ s.

**Figure 3:**
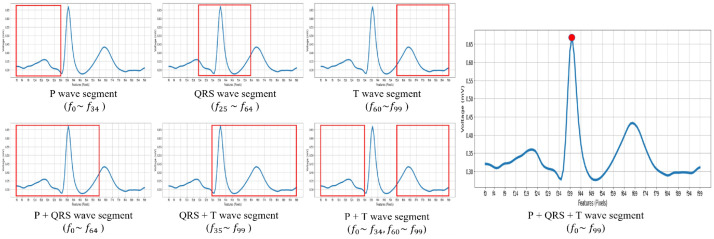
Schematic illustration of ECG heartbeat segment extraction and wave-segment division.

In other words, this study uniformly transforms each heartbeat cycle into a fixed-length time series of approximately 0.8 s with 100 sampling points. This setting ensures that different heartbeat samples have a consistent input dimensionality while preserving the major ECG waveform information before and after the R peak, which serves as the input basis for the subsequent time-series classification model. Figure 3 illustrates the extraction of ECG heartbeat segments based on the R peak and further marks the corresponding positions of the P wave, QRS complex, and T wave.

Based on the heartbeat segmentation procedure described above, the heartbeat-level dataset of the *s*-th patient follows the notation defined in Equation (1).

In this definition, $y_{i}^{\left(s\right)}\in \left\{0,1\right\}$ denotes the serum potassium status label corresponding to the *i*-th heartbeat segment, where 0 represents normokalemia and 1 represents serum potassium abnormality. Through this formulation, the original continuous ECG signal is represented as time-series classification data at the individual-heartbeat level, enabling the subsequent model to determine the serum potassium status for each heartbeat cycle.

After heartbeat segmentation, this study divides each heartbeat waveform into three major wave segments based on clinical physicians’ experience and further constructs seven types of wave-segment combination data.

Let the subsequences corresponding to the P wave, QRS complex, and T wave within the complete heartbeat segment $X_{i}^{\left(s\right)}$ of the *i*-th sample from the *s*-th patient be denoted by $X_{i,P}^{\left(s\right)}$, $X_{i,\mathrm{QRS}}^{\left(s\right)}$, and $X_{i,T}^{\left(s\right)}$, respectively. Here, $X_{i,P}^{\left(s\right)}$ denotes the P-wave segment in the *i*-th heartbeat sample, $X_{i,\mathrm{QRS}}^{\left(s\right)}$ denotes the QRS-complex segment, and $X_{i,T}^{\left(s\right)}$ denotes the T-wave segment. Since waveform lengths may vary across patients and across heartbeats, this study represents the construction of each wave-segment dataset through a wave-segment extraction and combination function, so as to provide a unified definition for the seven wave-segment inputs.

The seven wave segments follow the set B defined in Equation (2), where the symbol + denotes the combination or concatenation of wave segments rather than the numerical addition of signal values. For example, P+QRS denotes the wave-segment input formed jointly by the P wave and the QRS complex, whereas QRS+T denotes the wave-segment input formed jointly by the QRS complex and the T wave. For any wave segment b∈B, the input of the *i*-th heartbeat sample from the *s*-th patient under wave segment *b* can then be expressed as

(6) $$X_{i}^{\left(s,b\right)}=P_{b}\left(X_{i}^{\left(s\right)}\right)$$

Here, $P_{b}\left(\;\cdot \;\right)$ denotes the transformation function that extracts or combines the complete heartbeat segment according to wave segment *b*. When b=P, b=QRS, or b=T, it denotes extracting only the P-wave segment, the QRS-complex segment, or the T-wave segment, respectively. When b=P+QRS, b=QRS+T, or b=P+T, it denotes combining the corresponding two wave segments. When b=P+QRS+T, it denotes using the complete combination formed by the three major wave segments.

Accordingly, for the *s*-th patient and any wave segment *b*, the corresponding wave-segment dataset can be constructed as

(7) $$D^{\left(s,b\right)}={\left\{\left(X_{i}^{\left(s,b\right)},y_{i}^{\left(s\right)}\right)\right\}}_{i=1}^{N_{s}}$$

where $D^{\left(s,b\right)}$ denotes the time-series classification dataset formed for the *s*-th patient under wave segment *b*. Since each wave segment retains the same label $y_{i}^{\left(s\right)}$, the differences among the wave-segment datasets arise primarily from differences in the input waveform segments rather than changes in the classification target. Through this setting, this study can separately evaluate the classification capability of the P wave, QRS complex, T wave, and their different combinations in the serum potassium abnormality detection task.

### 4.2. Training of the Optimal Seven-Wave-Segment Submodels and SHAP Feature Augmentation

After constructing the seven ECG wave-segment datasets, this study further trains classification submodels separately for each ECG wave segment of each patient. Since different ECG wave segments reflect distinct physiological meanings and signal morphologies, a single model may not achieve the best classification performance across all wave segments. Therefore, this study adopts a strategy of independent multi-segment training and multi-model comparison. Submodels are constructed separately for the seven wave-segment datasets, and the best model for each wave segment is selected based on validation performance, serving as the basis for subsequent SHAP feature generation.

For each wave-segment dataset $D^{\left(s,b\right)}$ defined in Equation (7), this study trains multiple candidate time-series classification models, including MLP, FCN, TimeCNN [20], T-LeNet [21], ResNet [22], MCDCNN [23], and Encoder [24]. The set of candidate models is defined as

(8) $$A=\left\{\mathrm{MLP},\mathrm{FCN},\mathrm{TimeCNN},T\text{-}\mathrm{LeNet},\mathrm{ResNet},\mathrm{MCDCNN},\mathrm{Encoder}\right\}$$

For any candidate model a∈A after it is trained on the *b*-th wave segment of the *s*-th patient, it can be represented as

(9) $$f_{a,b}^{\left(s\right)}:X_{i}^{\left(s,b\right)}\to {\hat{p}}_{i,a,b}^{\left(s\right)}$$

where ${\hat{p}}_{i,a,b}^{\left(s\right)}$ denotes the predicted probability of serum potassium abnormality output by candidate model *a* based on the wave-segment input $X_{i}^{\left(s,b\right)}$. To select the most suitable submodel for each wave segment, this study uses validation-set AUC as the primary criterion for model selection, while F1-score and F2-score are also included as auxiliary evaluation metrics. The optimal candidate model for the *s*-th patient under wave segment *b* is defined as

(10) $$a_{b}^{*(s)}=\arg\max_{a\in A}{\mathrm{AUC}}_{\mathrm{val}}\left(f_{a,b}^{\left(s\right)}\right)$$

and its corresponding model is denoted as

(11) $$f_{b}^{*(s)}=f_{a_{b}^{*(s)},b}^{\left(s\right)}$$

where $a_{b}^{*(s)}$ denotes the optimal model type selected for the *s*-th patient under wave segment *b*, and $f_{b}^{*(s)}$ denotes the Best Learner of that patient for that wave segment. After model selection, each patient obtains seven optimal wave-segment submodels. For the *i*-th heartbeat sample, the outputs of the seven wave-segment Best Learners can be integrated into a seven-dimensional prediction vector:

(12) $$\begin{array}{cc} p_{i}^{\left(s\right)} & =\;[{\hat{p}}_{i,P}^{\left(s\right)},{\hat{p}}_{i,\mathrm{QRS}}^{\left(s\right)},{\hat{p}}_{i,T}^{\left(s\right)},{\hat{p}}_{i,P+\mathrm{QRS}}^{\left(s\right)},\\ & \;\;\;{\hat{p}}_{i,\mathrm{QRS}+T}^{\left(s\right)},{\hat{p}}_{i,P+T}^{\left(s\right)},{\hat{p}}_{i,P+\mathrm{QRS}+T}^{\left(s\right)}] \end{array}$$

where

(13) $${\hat{p}}_{i,b}^{\left(s\right)}=f_{b}^{*(s)}\left(X_{i}^{\left(s,b\right)}\right),\;b\in B$$

**Figure 4:**
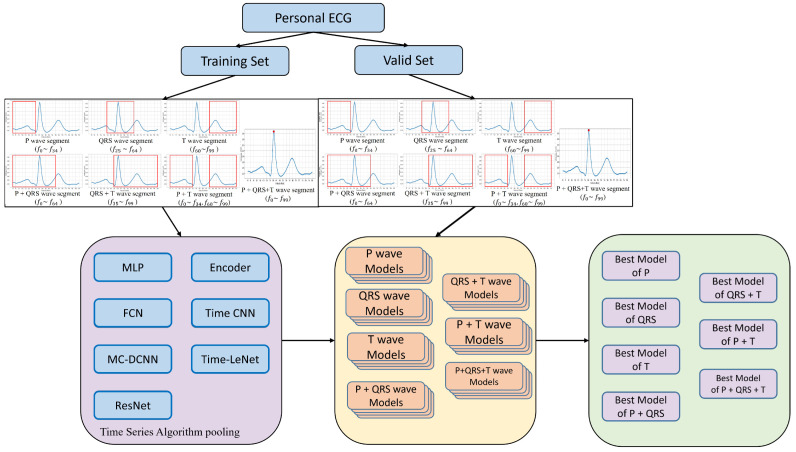
Training of seven-wave-segment candidate models and optimal submodel selection process.

This seven-dimensional vector $p_{i}^{\left(s\right)}$ represents the prediction results obtained from the seven wave-segment Best Learners for the same heartbeat sample. In other words, after the original ECG wave-segment time series are transformed by the corresponding wave-segment Best Learners, a set of high-level predictive features based on wave-segment model outputs is formed, serving as the basis for subsequent SHAP feature augmentation and ensemble classification. The overall process of seven-wave-segment candidate model training, validation evaluation, and optimal submodel selection is shown in Figure 4.

After training the seven wave-segment Best Learners, this study further applies the SHAP method to calculate the corresponding Shapley values for the seven-dimensional wave-segment prediction vector $p_{i}^{\left(s\right)}$ of each sample. The purpose of this stage is not to regenerate ECG signals, but to transform the prediction outputs of the seven wave-segment Best Learners into reusable feature representations. For the *i*-th sample, this study calculates the SHAP value of each of the seven wave-segment prediction features and forms the SHAP feature vector:

(14) $$\begin{array}{cc} \phi_{i}^{\left(s\right)} & =\;[\phi_{i,P}^{\left(s\right)},\phi_{i,\mathrm{QRS}}^{\left(s\right)},\phi_{i,T}^{\left(s\right)},\phi_{i,P+\mathrm{QRS}}^{\left(s\right)},\\ & \;\;\;\phi_{i,\mathrm{QRS}+T}^{\left(s\right)},\phi_{i,P+T}^{\left(s\right)},\phi_{i,P+\mathrm{QRS}+T}^{\left(s\right)}] \end{array}$$

where $\phi_{i}^{\left(s\right)}$ has the same dimensionality as the seven-dimensional wave-segment prediction vector $p_{i}^{\left(s\right)}$, but the two have different meanings. $p_{i}^{\left(s\right)}$ represents the prediction outputs of the seven wave-segment Best Learners for serum potassium abnormality, whereas $\phi_{i}^{\left(s\right)}$ represents the marginal contributions of these wave-segment prediction outputs to the prediction result of the SHAP source model.

To avoid label leakage during SHAP feature generation, this study adopts 5-fold cross-validation to generate out-of-fold SHAP features. Specifically, for the training data of each patient, the samples are divided into five mutually exclusive subsets. In each fold, only four subsets are used to train the SHAP source model, and SHAP values are calculated for the remaining subset that is not involved in training. Therefore, the SHAP features of each training sample are generated by a model that has not seen that sample, thereby reducing the risk of data leakage during feature generation. After completing the five-fold cross-validation, the SHAP features generated from the validation samples in each fold are integrated according to the sample indices, yielding the SHAP-augmented feature dataset covering the entire training data:

(15) $$D_{\mathrm{SHAP}}^{\left(s\right)}={\left\{\left(\phi_{i}^{\left(s\right)},y_{i}^{\left(s\right)}\right)\right\}}_{i=1}^{N_{s}}$$

where $D_{\mathrm{SHAP}}^{\left(s\right)}$ denotes the SHAP feature dataset generated for the *s*-th patient through the out-of-fold mechanism. This procedure ensures that the SHAP feature of each sample is generated by a model that was not trained on that sample, allowing the features to reflect the model’s decision behavior on unseen data and reducing the risk of label leakage during feature generation. In implementation, this study uses TreeExplainer to calculate Shapley values for tree-based and gradient boosting models. For non-tree-based models, KernelExplainer is used for estimation, so as to maintain consistency in the definition and interpretive meaning of SHAP features across different model architectures.

**Figure 5:**
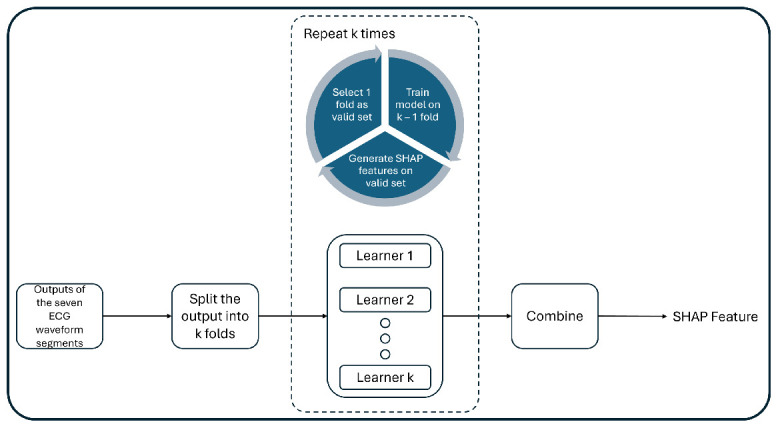
Schematic illustration of the SHAP feature generation process.

In summary, this study first trains candidate time-series classification models separately for the seven ECG wave segments and selects the Best Learner for each wave segment based on validation-set AUC. The prediction outputs of the seven Best Learners are then integrated into a seven-dimensional wave-segment prediction vector, and the corresponding SHAP feature vector is generated through 5-fold out-of-fold cross-validation. The feature generation process is illustrated in Figure 5. This procedure further transforms the prediction results of ECG wave-segment submodels into reusable augmented features with model-decision contribution information, serving as the input basis for subsequent stacking ensemble classification.

### 4.3. Final Ensemble Model Training Stage

After selecting the Best Learners for the seven ECG wave segments and generating SHAP features, this study further adopts stacking ensemble learning [25] as the final classification framework. The core concept of stacking is that multiple base learners in the first layer first generate prediction outputs, which are then transformed into input features for the second-layer classifier. This allows the meta-learner to integrate decision signals provided by different models or different data representations. Compared with a single model, stacking can exploit the complementarity among multiple model outputs, thereby reducing instability caused by the bias of a single wave segment or a single model and improving overall classification performance.

In this study, the first layer is not composed of conventional tabular-data classifiers, but rather consists of the Best Learners corresponding to the seven ECG wave segments. Specifically, in the previous stage, candidate time-series classification models were trained separately for the seven wave segments, and the optimal model for each segment was selected based on validation-set AUC, forming seven wave-segment-level base learners. Subsequently, the prediction outputs of the seven Best Learners were integrated into the seven-dimensional wave-segment prediction vector $p_{i}^{\left(s\right)}$, and the corresponding SHAP feature vector $\phi_{i}^{\left(s\right)}$ was further generated through the 5-fold out-of-fold mechanism. Therefore, this stage does not redefine the wave-segment model outputs, but directly uses the previously defined $p_{i}^{\left(s\right)}$ and $\phi_{i}^{\left(s\right)}$ as the input basis for the final stacking classifier.

To examine the practical effectiveness of SHAP features in the final classification stage, this study designs three stacking input settings: Baseline, SHAP+Original, and SHAP-only. Let *u* denote the input setting. The final model input for the *i*-th heartbeat sample of the *s*-th patient under different input settings can be defined as

(16) $$h_{i}^{\left(s,u\right)}=\left\{\begin{array}{cc} p_{i}^{\left(s\right)}, & u=\mathrm{Baseline},\\ \left[p_{i}^{\left(s\right)};\phi_{i}^{\left(s\right)}\right], & u=\text{SHAP+Original},\\ \phi_{i}^{\left(s\right)}, & u=\text{SHAP-only}. \end{array}\right.$$

where $h_{i}^{\left(s,u\right)}$ denotes the input feature of the final classifier formed for the *i*-th sample of the *s*-th patient under input setting *u*, and $\left[\;\cdot \;;\;\cdot \;\right]$ denotes vector concatenation. When u=Baseline, the model uses only the original prediction outputs of the seven wave-segment Best Learners. When u= SHAP+Original, the model simultaneously uses the seven-dimensional wave-segment prediction vector and the seven-dimensional SHAP feature vector, resulting in an input dimension of 14. When u=SHAP-only, the model uses only SHAP features as input, so as to examine whether SHAP features are capable of serving as an independent feature representation. This design enables separate evaluation of the classification effectiveness of the original wave-segment model outputs, SHAP-augmented features, and SHAP-based alternative representations in the serum potassium abnormality detection task.

For the second-layer classifier, this study employs four machine learning models as stacking meta-learners, including Logistic Regression [26], Support Vector Machine (SVM) [27], k-Nearest Neighbors (KNN) [28], and Categorical Boosting (CatBoost) [29], in order to compare the adaptability of different classification mechanisms to the three input settings. Logistic Regression integrates input features through linear weighting and offers relatively high interpretability, making it suitable as a baseline classifier. SVM can construct classification boundaries and handle nonlinear data distributions. KNN performs classification based on sample neighborhood relationships, allowing the discriminative ability of different feature representations in the sample space to be examined. CatBoost can capture nonlinear relationships and interactions among features. Through the comparison of these four meta-learners, this study further analyzes the stability and applicability of SHAP features under different classification mechanisms.

After constructing features under the three input settings, this study trains the corresponding final stacking classification model for each meta-learner and each input setting. Let *m* denote the adopted meta-learner, and let $u\in \left\{\mathrm{Baseline},\;\text{SHAP+Original},\;\text{SHAP-only}\right\}$ denote the input setting. The final stacking classification model of the *s*-th patient can be expressed as

(17) $$H_{m,u}^{\left(s\right)}:h_{i}^{\left(s,u\right)}\to {\hat{q}}_{i,m,u}^{\left(s\right)}$$

where $H_{m,u}^{\left(s\right)}$ denotes the final ensemble model constructed for the *s*-th patient under meta-learner *m* and input setting *u*, and ${\hat{q}}_{i,m,u}^{\left(s\right)}\in \left[0,1\right]$ denotes the predicted probability that the *i*-th heartbeat sample belongs to the serum potassium abnormality class. In other words, the second-layer classifier does not directly process the raw ECG time series. Instead, it learns from the high-level predictive features formed by the seven wave-segment Best Learners in the first layer and the contribution features transformed by SHAP, thereby producing the final prediction result for serum potassium abnormality.

To further obtain binary classification results, this study converts the predicted probability of serum potassium abnormality into a class label according to the classification threshold τ:

(18) $${\hat{y}}_{i,m,u}^{\left(s\right)}=\left\{\begin{array}{cc} 1, & {\hat{q}}_{i,m,u}^{\left(s\right)}\geq \tau ,\\ 0, & {\hat{q}}_{i,m,u}^{\left(s\right)}<\tau . \end{array}\right.$$

Here, ${\hat{y}}_{i,m,u}^{\left(s\right)}=1$ indicates that the model classifies the heartbeat sample as serum potassium abnormality, whereas ${\hat{y}}_{i,m,u}^{\left(s\right)}=0$ indicates that the model classifies the heartbeat sample as normokalemia. τ denotes the classification decision threshold. Through this definition, the final stacking model can convert the predicted probabilities under different input settings into actual classification results, which are then used for subsequent performance evaluation.

### 4.4. Overall Modeling Framework and Evaluation Metrics

Accordingly, the overall data flow of this study can be conceptually represented as follows:

(19) $$X_{i}^{\left(s\right)}\to {\left\{X_{i}^{\left(s,b\right)}\right\}}_{b\in B}\to p_{i}^{\left(s\right)}\to \phi_{i}^{\left(s\right)}\to h_{i}^{\left(s,u\right)}\to H_{m,u}^{\left(s\right)}\to {\hat{q}}_{i,m,u}^{\left(s\right)}$$

where ${\left\{X_{i}^{\left(s,b\right)}\right\}}_{b\in B}$ denotes the seven wave-segment representations corresponding to the *i*-th ECG heartbeat sample of the *s*-th patient, $p_{i}^{\left(s\right)}$ denotes the seven-dimensional wave-segment prediction vector formed by the outputs of the seven wave-segment Best Learners, $\phi_{i}^{\left(s\right)}$ denotes the SHAP feature vector representing the marginal contributions of the seven wave-segment prediction outputs in $p_{i}^{\left(s\right)}$ to the prediction result of the SHAP source model, and $h_{i}^{\left(s,u\right)}$ denotes the final input feature under input setting *u*. In addition, $H_{m,u}^{\left(s\right)}$ denotes the final stacking ensemble classifier constructed under meta-learner *m* and input setting *u*, and ${\hat{q}}_{i,m,u}^{\left(s\right)}$ denotes the predicted probability that the *i*-th sample belongs to the serum potassium abnormality class.

For the performance evaluation of the final stacking classification model, Precision, Recall, AUC, F1-score, and G-Mean are used as evaluation metrics. Precision measures the proportion of samples predicted as serum potassium abnormality that are truly abnormal. Recall measures the model’s ability to detect serum potassium abnormality samples. AUC evaluates the overall discriminative ability of the model across different thresholds. F1-score jointly considers Precision and Recall, whereas G-Mean reflects the model’s balanced recognition ability across different classes. Through these metrics, this study can simultaneously examine model discrimination, minority-class detection ability, and classification stability, and compare the performance differences among the Baseline, SHAP+Original, and SHAP-only input settings under different meta-learners.

Overall, the stacking architecture in this study can be regarded as a two-layer classification process. In the first layer, the seven ECG wave-segment Best Learners separately extract decision information related to serum potassium abnormality from different ECG wave segments and form the aforementioned seven-dimensional wave-segment prediction vector. In the second layer, meta-learners are trained using the three input settings, namely Baseline, SHAP+Original, and SHAP-only, to generate the final prediction result for serum potassium abnormality. Through this design, this study systematically compares the effects of original wave-segment model outputs, SHAP-augmented features, and SHAP-based alternative feature representations on final classification performance, and further validates the effectiveness and interpretive value of SHAP feature augmentation in ECG-based serum potassium abnormality detection.

## 5. Experimental Results

### 5.1. Dataset Description

The ECG data used in this study were obtained from the MIMIC-III database [30], formally known as the Medical Information Mart for Intensive Care III. The data consist of continuous one-dimensional time-series signals. Since ECG data are characterized by inter-patient variability, differences in measurement time, and inconsistent signal quality, failure to perform data organization and label alignment in advance may lead to incorrect sample correspondence or unstable classification results during model training.

Regarding temporal information, each ECG record contains two types of time information: Time ID and Chart Time. Time ID is used to indicate the hospitalization period to which the patient belongs, whereas Chart Time corresponds to the specific measurement time of blood sampling, laboratory testing, or related clinical records. Since the same patient may have multiple Chart Time records during the same hospitalization period, this study pairs ECG signals with their corresponding serum potassium test results based on both Time ID and Chart Time, ensuring that each ECG sample has a clear and consistent classification label. If a patient has multiple records under the same Time ID, these records are regarded as repeated measurements under the same hospitalization status. If the records belong to different Time IDs, they represent different hospitalization periods or clinical states and must therefore be processed separately to avoid confusing physiological conditions from different time periods.

Since the patient’s physiological condition usually does not change substantially within the same Time ID, the ECG data collected under the same Time ID are generally less variable. If data from the same Time ID are used simultaneously for model training and testing, the model may obtain better prediction results. To avoid using data from the same Time ID in both the training and testing sets, the testing set in this study was constructed using Time IDs different from those used in the training and validation sets. This setting reduces the possibility that similar ECG samples from the same hospitalization period appear simultaneously in the training and testing stages.

After completing the above data organization, temporal alignment, label pairing, and Time ID-based partitioning procedures, this study summarizes the ECG samples of eight patients in the training, validation, and test sets. The total number of samples in each dataset, as well as the numbers of normokalemic and serum potassium abnormal samples, are shown in Table 1.

### 5.2. Experimental Results and Analysis

#### 5.2.1. Optimal Model Selection Results for ECG Wave-Segment Submodels

Since ECG signal morphology varies across patients, this study trains corresponding submodels separately for each patient across seven wave segments, including P, QRS, T, P+QRS, QRS+T, P+T, and P+QRS+T. The performance of each wave-segment model is evaluated using AUC, F1-score, and F2-score. Although the optimal models corresponding to different evaluation metrics are not entirely consistent for some patients, most wave segments still exhibit similar model-selection trends. To maintain consistency in the subsequent analysis procedure and feature augmentation process, this study uses AUC as the primary criterion for model selection to determine the optimal submodel for each patient under each wave segment. The optimal model selection results for the eight patients across the seven wave segments are shown in Table 2.

As observed from Table 2, Encoder is the most frequently selected architecture across patients and wave segments, followed by ResNet and T-LeNet. This result indicates that architectures with sequence-dependency modeling or residual representation learning show relatively stable adaptability to different ECG wave-segment inputs. In particular, Encoder appears repeatedly across multiple patients and wave segments, whereas ResNet is frequently selected in several composite wave-segment settings, especially for patient 60274.

Although MCDCNN is selected in several cases, it does not show a dominant overall selection frequency. Instead, its selections are concentrated in specific patients and composite wave segments, particularly QRS + T and P + QRS + T in patient 18996, as well as QRS + T and P + T in patient 65112. This pattern suggests that MCDCNN may capture discriminative information in certain patient-specific composite wave segments, rather than exhibiting a generally dominant selection pattern across all patients and wave segments. By contrast, FCN is mainly selected in several individual wave-segment cases, MLP appears in more isolated patient- and segment-specific settings, and TimeCNN is rarely selected. Overall, these results indicate that the optimal submodel varies across patients and ECG wave segments, while Encoder, ResNet, and T-LeNet exhibit relatively more frequent selection patterns in the overall comparison.

#### 5.2.2. Stacking Ensemble Results and SHAP Feature Augmentation Analysis

This study includes eight patients in the stacking ensemble classification experiment, and the detailed patient-level results are provided in Supplementary Table S1. To further examine the performance differences of SHAP feature augmentation across different patients and classifiers, patients 46092, 60274, and 75557 are selected as representative cases and analyzed together with their SHAP beeswarm results. Supplementary Table S1 compares the performance of four classifiers, namely Logistic Regression, SVM, KNN, and CatBoost, under three input settings: Baseline, SHAP+Original, and SHAP-only. The reported metrics include Precision, Recall, AUC, F1-score, and G-Mean. Through this representative case analysis, the performance variations and applicability conditions of SHAP feature augmentation under different patient-specific data characteristics and model structures can be more clearly examined.

**Figure 6:**
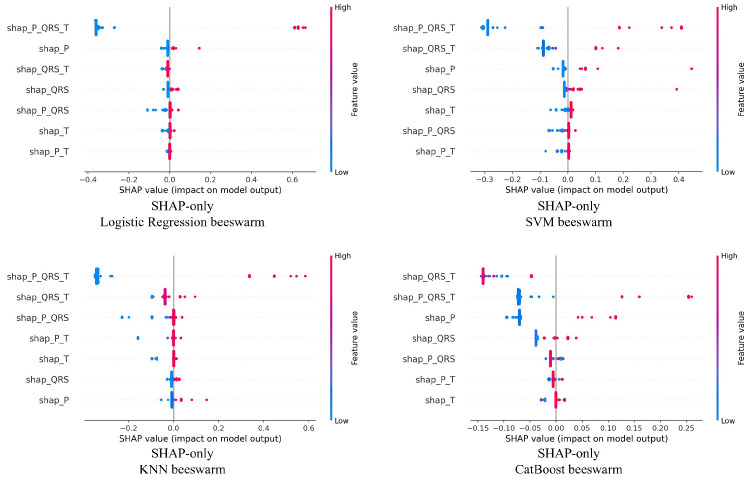
Beeswarm results of four classifiers under the SHAP-only setting for patient 46092.

Patient 46092 exhibits a relatively stable and general improvement trend. Logistic Regression, SVM, and CatBoost all perform better than or slightly better than Baseline under the SHAP-only setting. Among them, SVM achieves the most prominent performance for this patient, with the F1-score and G-Mean increasing to 0.8375 and 0.8749, respectively. However, although KNN achieves higher Precision under the SHAP-only setting, its Recall decreases to 0.3578, causing the F1-score and G-Mean to decline to 0.5036 and 0.5881, respectively. From the beeswarm results in Figure 6, it can be observed that all four classifiers regard P+QRS+T as the main contribution source, but their feature dependency patterns are not identical. For Logistic Regression, the contribution is mainly concentrated on P+QRS+T, while the remaining wave segments are mostly close to zero. For SVM, in addition to P+QRS+T, clearer auxiliary contributions are also observed in QRS+T and P, which is consistent with its best performance under the SHAP-only setting. Although KNN presents nonzero contributions across multiple wave segments, including P+QRS+T, QRS+T, P+QRS, and P+T, these contributions may not form stable neighborhood relationships in the distance-based classification structure, which may be related to the decline in its Recall and overall performance. CatBoost is mainly influenced by QRS+T, P+QRS+T, and P, and maintains relatively favorable classification results. Overall, the results of patient 46092 indicate that SHAP features have the potential to serve independently as discriminative inputs, but their effectiveness remains affected by the characteristics of the downstream classifier.

**Figure 7:**
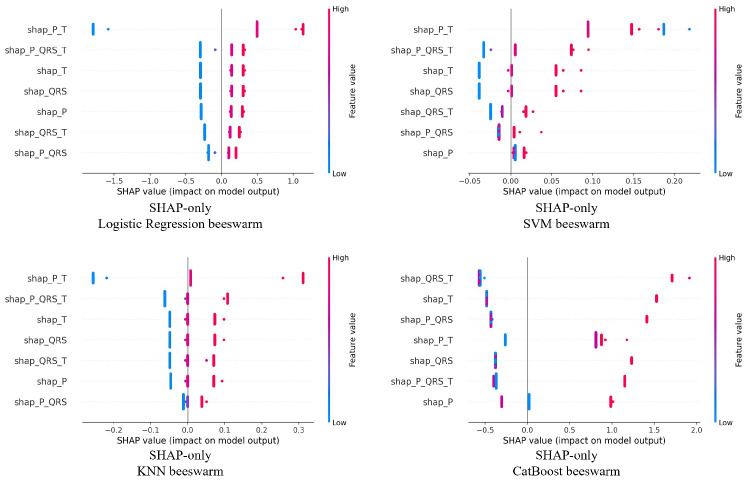
Beeswarm results of four classifiers under the SHAP-only setting for patient 60274.

The results of patient 60274 most clearly reflect the substitutability of the SHAP-only setting. Compared with Baseline and SHAP+Original, for which most models achieve an F1-score of approximately 0.6626 and a G-Mean of approximately 0.7691, SHAP-only achieves an F1-score and G-Mean of approximately 0.9982 across all four classifiers. This indicates that SHAP features retain highly discriminative information for this patient and can maintain nearly complete classification performance without relying on the original seven-wave-segment outputs. From the beeswarm results in Figure 7, although different classifiers exhibit different wave-segment dependency patterns, they all form clear feature contribution distributions. Logistic Regression and SVM mainly use P+T and P+QRS+T as the primary discriminative sources, whereas KNN is simultaneously influenced by P+T, P+QRS, and P+QRS+T. CatBoost shows more distinct feature separability for QRS+T, T, and P+QRS, with a wider distribution range of SHAP values. Overall, the results of patient 60274 show that all classifiers maintain stable and highly consistent classification performance under the SHAP-only setting, further supporting the use of SHAP features as an effective alternative representation for the original outputs.

**Figure 8:**
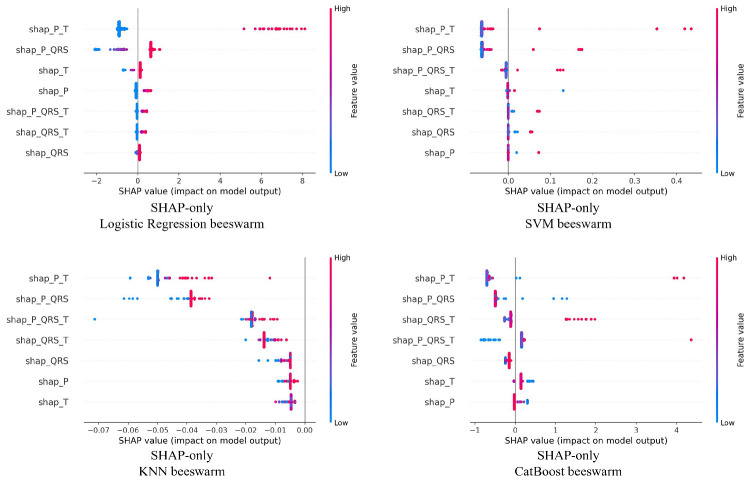
Beeswarm results of four classifiers under the SHAP-only setting for patient 75557.

Patient 75557 further demonstrates the clear model dependency of SHAP feature augmentation. Logistic Regression and SVM both improve over Baseline under the SHAP-only setting, indicating that SHAP features still provide a certain degree of enhancement for linear or boundary-oriented models. By contrast, although KNN and CatBoost improve under the SHAP+Original setting, they degrade markedly under the SHAP-only setting. In particular, the F1-score of KNN decreases to 0.0014, while the Precision, Recall, F1-score, and G-Mean of CatBoost are all 0.0000. From the beeswarm results in Figure 8, Logistic Regression mainly shows more evident feature contributions from P+T, P+QRS, and T, whereas SVM primarily relies on P+T, P+QRS, and P+QRS+T, indicating that some wave-segment SHAP features can still provide effective discriminative information. However, the SHAP values of KNN are mostly concentrated in the negative or near-zero regions, suggesting that it fails to form a stable and separable feature contribution pattern under the SHAP-only setting. Although CatBoost shows local contributions in P+T, P+QRS, QRS+T, and P+QRS+T, the distribution is relatively scattered and fails to produce effective classification results under the predefined classification threshold. These results indicate that, when fully relying on SHAP features, tree-based models may not be able to reproduce the decision structure formed based on the original outputs, leading to classification failure under the predefined threshold. Overall, the results of patient 75557 indicate that SHAP features cannot independently replace the original outputs for all classifiers, and their effectiveness remains highly influenced by model structure and the stability of feature distributions.

Taken together, the overall results of the eight patients in Supplementary Table S1 indicate that, after SHAP features are incorporated into the final stacking input, classification performance can be maintained or improved in most patients and classifiers. In particular, SHAP-only also achieves better F1-score, AUC, or G-Mean than the Baseline in some patients, suggesting its potential to assist the original outputs and provide alternative representations. However, the effectiveness of SHAP features is not consistent across all patients and classifiers. Among the representative patients selected in this study, patient 60274 achieves the best performance under the SHAP-only setting, indicating that SHAP features can highly preserve the discriminative information of the seven-wave-segment submodels. Patient 46092 shows that SHAP features have a positive effect on most classifiers, although KNN exhibits degraded performance under the SHAP-only setting. Patient 75557 further demonstrates that SHAP+Original can improve the performance of some models, whereas SHAP-only clearly fails in KNN and CatBoost. Overall, SHAP features can not only serve as interpretable high-level augmented representations but also demonstrate the potential to reduce model dependence on the original output features under specific conditions. These findings support the concept of using SHAP features as alternative representations for retraining, and provide a feasible research direction for future cross-institutional model sharing, privacy-preserving learning, and model generalization.

Table 3 summarizes the average stacking performance of the eight patients under different final classifiers and input settings. The cross-patient mean F1-score and G-Mean are reported to compare the four final classifiers under the three input settings. The results show that, after introducing SHAP features, most models outperform the Baseline, indicating that SHAP features can effectively complement the discriminative information provided by the outputs of the seven-wave-segment submodels.

Among the models, Logistic Regression shows the most pronounced improvement when only SHAP features are used. Its average F1-score increases from 0.594 to 0.785, and its G-Mean increases from 0.561 to 0.728, indicating that SHAP features themselves have strong classification representativeness. SVM and KNN also achieve higher F1-scores under the SHAP-only setting, although their G-Mean slightly decreases. This result suggests that SHAP-only features still retain useful discriminative information, while their effects may vary across classifiers. In addition, the SHAP+Original setting shows a relatively consistent improvement trend across classifiers, indicating that combining SHAP features with the original wave-segment outputs can further support final classification. CatBoost achieves its best performance under the SHAP+Original setting, with the F1-score increasing from 0.595 to 0.659 and the G-Mean increasing from 0.575 to 0.634.

#### 5.2.3. Performance Analysis of SHAP Feature Sources and Training Data Size

To analyze the influence of the generation conditions of SHAP-augmented features on final classification performance, this study further compares two factors: first, SHAP features generated from different source models; and second, the proportion of training data used to generate SHAP features. To avoid the influence of downstream classifier differences on result interpretation, this experiment fixes Logistic Regression as the final classifier and separately evaluates the effects of SHAP source models and training data size on Precision, Recall, AUC, F1-score, and G-Mean.

Supplementary Table S2 summarizes the classification results of the eight patients under different SHAP source models. Overall, when CatBoost is used as the SHAP source model, it shows a higher performance ceiling in most patients, particularly in 13593, 60274, 65112, 75557, and 83013, where F1-score, AUC, or G-Mean is clearly improved. Taking 60274 as an example, the F1-score of the Baseline and the SHAP features generated from LR, SVM, and KNN all remain at 0.6626, whereas CatBoost-SHAP increases the F1-score to 0.9982. Similarly, in 65112, the F1-score increases from 0.6700 in the Baseline to 0.9847, indicating that nonlinear source models can generate more discriminative SHAP-augmented features for some patient-specific data.

However, CatBoost-SHAP does not consistently improve all metrics across all patients. In 46092, although CatBoost-SHAP increases Precision to 0.9948 and AUC to 0.9011, Recall decreases to 0.3584, causing F1-score and G-Mean to decline to 0.5270 and 0.5984, respectively. In 75350, F1-score improves, but G-Mean decreases to 0.0000, suggesting that the classification results may become biased toward a single class. By contrast, 18996 shows only minimal performance differences among different SHAP source models, indicating that the influence of the SHAP source model is strongly patient-dependent.

Supplementary Table S3 further compares the performance of LR-SHAP features generated under different training data proportions. The results show that increasing the amount of training data used for the SHAP source model does not lead to consistent improvement across all patients, but instead results in different trends depending on patient-specific data characteristics. The improvement is most evident in 46092. When the training proportion increases from 10% to 100%, the F1-score increases from 0.5312 to 0.6991, and the G-Mean also increases from 0.6232 to 0.7597, indicating that a higher training proportion helps generate more stable SHAP features.

By contrast, the F1-score and G-Mean of 18996, 60274, 65112, and 83013 remain nearly unchanged under different training proportions, suggesting that increasing the data size has limited impact on the effectiveness of their LR-SHAP features. For 13593, the improvement mainly occurs between the 10% and 30% training proportions, after which the performance tends to stabilize. For 75350, better performance is observed under the 30% or 50% training proportion, whereas the performance slightly decreases at 100%, indicating that a larger amount of training data does not necessarily yield the best results for all patients.

Taken together, Supplementary Tables S2 and S3 show that the classification performance of SHAP-augmented features is jointly affected by the source model and the amount of data used for feature generation. Among them, CatBoost-SHAP demonstrates greater improvement potential in most patients, but it may also introduce trade-offs among Precision, Recall, and G-Mean. Increasing the training data size leads to clear improvement only in some patients and does not produce a consistent linear enhancement. These results indicate that SHAP-augmented features are not universally effective; their actual performance should be evaluated jointly based on the source model, data-size condition, and patient-specific data characteristics.

#### 5.2.4. Comparison of Classification Performance Between PCA- and SHAP-Based Feature Augmentation

To compare the effects of statistical dimensionality reduction and model-driven feature representation on classification performance, this study evaluates PCA and SHAP features under two settings. The first setting combines the transformed features with the original inputs, namely PCA+Original and SHAP+Original. The second setting retains only the transformed features, namely PCA-only and SHAP-only. PCA primarily preserves the directions of linear variance in the data, whereas SHAP reflects the decision contribution of input features to the model. Therefore, the two methods represent statistical-oriented and model-interpretation-oriented feature representation approaches, respectively. To avoid comparison bias caused by a fixed number of principal components, this study selects the optimal PCA setting based on the validation performance of each patient and each final classifier across different PCA dimensional settings. In contrast, SHAP features preserve the complete contribution information of the seven wave-segment prediction outputs to the model decision.

Supplementary Table S4 summarizes the classification results of eight patients under the Baseline, PCA+Original, and SHAP+Original settings. To further compare the effects of PCA- and SHAP-based feature augmentation on classification performance, patients 13593, 18996, and 75350 are selected as representative cases for analysis. As observed from Supplementary Table S4, SHAP+Original is more capable of maintaining or improving model performance in some patients. Taking patient 13593 as an example, PCA+Original reduces the F1-score and G-Mean of Logistic Regression from 0.4186 and 0.5743 to 0.0263 and 0.1414, respectively. By contrast, SHAP+Original maintains the same F1-score and G-Mean, and further achieves 0.4394 and 0.6193 with CatBoost, outperforming PCA+Original. Patient 18996 shows more stable results, where SHAP+Original achieves F1-score/G-Mean values of 0.7135/0.7201 and 0.7184/0.7281 for SVM and KNN, respectively, both slightly higher than those of PCA+Original. Conversely, patient 75350 indicates that PCA+Original has greater advantages. For example, the G-Mean of Logistic Regression increases from 0.2465 to 0.6598, and that of KNN also increases from 0.3168 to 0.6360, whereas SHAP+Original does not provide the same degree of improvement. These results indicate that SHAP features can provide effective auxiliary information in most cases with stable decision signals, while PCA still offers feature restructuring effects in some cases with more specific data distributions.

Supplementary Table S5 summarizes the classification results of eight patients under the Baseline, PCA-only, and SHAP-only settings, aiming to examine the information retention capability of PCA and SHAP features when they are not combined with the original outputs. To further illustrate the differences between the two feature representation approaches across different patients and classifiers, patients 13593, 18996, and 75350 are selected as representative cases for analysis. As observed from Supplementary Table S5, SHAP-only demonstrates greater substitution potential in most representative results. Taking patient 13593 as an example, PCA-only reduces the F1-score/G-Mean of Logistic Regression to 0.0276/0.1468, whereas SHAP-only increases them to 0.9342/0.9790. For KNN, the F1-score/G-Mean also increase from 0.2765/0.4481 in the Baseline to 0.4624/0.6758. The results for patient 18996 are even more consistent: SHAP-only achieves 0.9829/0.9895 with KNN and 0.8328/0.8714 with CatBoost, both outperforming the Baseline and PCA-only settings. Although PCA-only shows greater advantages in classification balance for patient 75350, suggesting that PCA may still be applicable to certain data structures, the overall results indicate that SHAP features better preserve the key decision information learned during model training when used independently.

Taken together, Supplementary Tables S4 and S5 indicate that PCA and SHAP exhibit different feature representation characteristics. PCA compresses data variability through linear principal components and can improve the structure of the feature space in some patients and classifiers, particularly showing better classification balance in patient 75350. However, its representation is mainly derived from the statistical variation of the data itself and does not directly reflect the model’s decision logic; therefore, its ability to retain information remains limited under some settings. In contrast, SHAP features are generated based on internal model contributions, making them more closely aligned with the model’s actual decision basis.

From the overall results of the eight patients, SHAP+Original can maintain or improve the Baseline performance in most patients and classifiers, while SHAP-only also demonstrates better information retention capability than PCA-only in most settings. Among them, patients 60274, 65112, 46092, and 18996 show more evident performance preservation or substitution potential using SHAP features. However, the effectiveness of SHAP features is not consistent across all patients and classifiers. For example, patient 75350 shows better G-Mean under the PCA-only setting, while some classifiers for patients 75557 and 13593 also exhibit performance degradation under the SHAP-only setting.

## 6. Discussion

The experimental results show that SHAP-based feature augmentation can provide effective auxiliary information for ECG-based serum potassium abnormality prediction. From the overall results of the eight patients, incorporating SHAP features into the final stacking input generally maintained or improved the classification performance of most models. In particular, for Logistic Regression, the SHAP-only setting achieved a notable improvement, with the average F1-score increasing from 0.594 in the Baseline setting to 0.785, and the G-Mean increasing from 0.561 to 0.728. For CatBoost, the best performance was obtained under the SHAP+Original setting, where the F1-score increased from 0.595 to 0.659 and the G-Mean increased from 0.575 to 0.634. These results indicate that SHAP features can supplement the discriminative information provided by the seven wave-segment submodel outputs and, in some settings, can also serve as an alternative feature representation.

Compared with previous ECG-based serum potassium abnormality detection studies, An et al. [6] mainly developed DeepECG-Hyperkalemia and DeepECG-Hypokalemia models for detecting hyperkalemia and hypokalemia, and analyzed the influence of different ECG wave segments on model prediction by masking the P wave, QRS complex, and T wave. Galloway et al. [17] developed a deep learning model based on multiple ECG leads for noninvasive hyperkalemia screening. In contrast, the present study does not focus on constructing an end-to-end deep learning screening model, but further investigates whether the SHAP features generated from the outputs of seven ECG wave-segment submodels can serve as augmented inputs and auxiliary feature representations for the downstream stacking classifier.

Compared with Chiu et al. [18], their study proposed a personalized hyperkalemia detection method using lead II ECG excerpts within 10 min before serum potassium measurement from the MIMIC-III Waveform Database Matched Subset. They first trained a general 1D ResNet model using data from a large number of patients, and then applied transfer learning and fine-tuning using individual patient data. Their results showed that the accuracy increased from 0.604 in the general model to 0.980 in the personalized model, and the AUC increased from 0.729 to 0.945. In comparison, the present study also considers individual differences in ECG-based serum potassium abnormality detection, but adopts a different methodological design. Chiu et al. [18] mainly improved the detection performance of the deep learning model through personalized fine-tuning, whereas the present study generates SHAP-based augmented features based on the outputs of seven ECG wave-segment submodels and compares three input settings, namely Baseline, SHAP+Original, and SHAP-only, to evaluate the effectiveness of model explanation information as reusable features.

Due to strict restrictions under relevant regulations in Taiwan regarding the use and external provision of clinical data, raw clinical data generally cannot be taken outside the hospital environment in practical research workflows. Therefore, if augmented feature columns that do not directly contain the original signal values can be generated from ECG waveform data within the hospital and used as inputs for downstream classification models, the dependence on the external transfer of raw data can be reduced. Based on this motivation, the present study further investigates whether SHAP-based augmented features can preserve sufficient discriminative information and serve as auxiliary feature representations for medical signal classification tasks.

On the other hand, the present study does not directly use a single ECG input for ensemble learning. Instead, each heartbeat sample is first transformed into seven wave-segment datasets, and the Best Learner for each wave segment is selected to generate prediction outputs. These seven wave-segment model outputs are then integrated into the downstream stacking classifier. Therefore, the present study focuses on combining multiple ECG wave-segment representations with their corresponding best models, and further evaluates the role of SHAP-based augmented features in the final classification task.

Overall, previous studies have demonstrated the relationship between ECG signals and serum potassium abnormality detection from the perspectives of ECG signal feasibility, wave-segment importance, large-scale clinical screening, and personalized transfer learning. Based on these findings, the present study further investigates this problem from the perspectives of data-use restrictions, multi-segment model integration, and SHAP-based feature augmentation. The experimental results show that, under some patient and classifier settings, SHAP features not only provide model decision explanations but also preserve useful discriminative information, allowing them to serve as auxiliary or alternative inputs for the final classification model. These results suggest that SHAP-based augmented features have potential as derived feature representations in restricted data-use settings and personalized ECG classification tasks.

## 7. Conclusions

This study proposes a classification framework for serum potassium abnormality detection in ECG time-series data by integrating seven ECG wave-segment submodels, SHAP feature augmentation, and stacking ensemble learning. Through a personalized modeling strategy, this study separately extracts signal features from the P, QRS, T, and combined wave segments for different patients, and incorporates SHAP values as augmented features into the final classification model to evaluate the application value of model-driven feature representations in medical time-series classification tasks.

The experimental results show that SHAP features can provide effective auxiliary classification information for most patients and classifiers. Even when only SHAP features are used, some models can still maintain or outperform the Baseline, indicating that SHAP features can preserve key discriminative information from the model decision-making process. However, the responses to SHAP features are not entirely consistent across different patients and classifiers, suggesting that their effectiveness remains influenced by patient-specific data distributions and model structures.

Further analysis of the generation conditions of SHAP-augmented features shows that their classification effectiveness varies with the source model and the amount of training data, and no single optimal setting consistently applies to all patients. Experiments with different training proportions also indicate that increasing the training data size can improve the quality of SHAP features for some patients, but the overall trend does not show a consistent linear increase. These findings suggest that SHAP feature augmentation does not depend solely on feature transformation itself; rather, its actual effectiveness is closely related to the learning capability of the source model, the data-size condition, and patient-specific data characteristics. Compared with PCA, SHAP features demonstrate better information retention and substitution potential in most representative results. PCA primarily restructures data variability based on linear principal components and can improve the feature space and classification balance for some patients, whereas SHAP is generated from model prediction contributions and therefore more directly reflects the model’s actual decision basis.

Overall, this study validates the feasibility of applying SHAP features as an explainable feature augmentation method to ECG time-series classification tasks. Its value lies not only in improving classification performance for some models and patient datasets but also in transforming model decision contributions into reusable synthetic feature representations, thereby enabling a better balance among performance, interpretability, and data reuse. In particular, under the SHAP-only setting, the model can maintain a certain level of classification ability without directly relying on the original ECG wave-segment outputs or raw signal data, indicating that such model-driven synthetic features have research potential as an alternative form of medical data sharing. These findings can serve as a basis for future privacy-preserving data sharing and cross-institutional model collaboration, and further demonstrate the application potential of SHAP features as synthetic feature representations. Future studies may continue to evaluate their privacy-preserving capability, cross-dataset generalizability, and clinical applicability under different patient data characteristics, source model structures, and training data-size conditions.

## Figures and Tables

**Table 1:** Distribution of normokalemic and serum potassium abnormal samples in the training, validation, and test sets of eight patients from MIMIC-III.

Patients (Subject ID)	Training Samples (Normal:Abnormal)	Validation Samples (Normal:Abnormal)	Testing Samples (Normal:Abnormal)
13593	1659 (896:763)	414 (224:190)	1462 (1175:287)
18996	682 (199:483)	169 (49:120)	691 (432:259)
46092	22,722 (13,786:8936)	5680 (3446:2234)	15,509 (10,152:5357)
60274	534 (387:147)	132 (96:36)	957 (683:274)
65112	23,404 (16,065:7339)	5846 (4012:1834)	17,801 (11,745:6056)
75350	6682 (1911:4771)	1663 (472:1191)	8884 (2933:5951)
75557	46,568 (39,726:6842)	11,642 (10,472:1710)	26,975 (22,534:4441)
83013	432 (212:220)	107 (53:54)	1040 (782:258)

**Table 2:** Optimal submodel selection results based on AUC, F1-score, and F2-score across seven ECG wave segments for eight patients from MIMIC-III.

Patient ID/ECG Waves	P	QRS	T	P + QRS	QRS + T	P + T	P + QRS + T
**13593**							
AUC	FCN	TimeCNN	FCN	MLP	Encoder	MLP	Encoder
F1-score	FCN	Encoder	FCN	Encoder	Encoder	Encoder	Encoder
F2-score	FCN	Encoder	FCN	Encoder	Encoder	Encoder	MLP
**18996**							
AUC	FCN	ResNet	MLP	MLP	MCDCNN	T-LeNet	MCDCNN
F1-score	FCN	ResNet	MLP	MLP	MCDCNN	T-LeNet	MCDCNN
F2-score	FCN	ResNet	MCDCNN	MLP	MCDCNN	T-LeNet	MCDCNN
**46092**							
AUC	Encoder	Encoder	Encoder	T-LeNet	Encoder	T-LeNet	Encoder
F1-score	Encoder	Encoder	Encoder	T-LeNet	Encoder	T-LeNet	Encoder
F2-score	T-LeNet	Encoder	Encoder	T-LeNet	Encoder	T-LeNet	Encoder
**60274**							
AUC	FCN	T-LeNet	T-LeNet	ResNet	ResNet	ResNet	Encoder
F1-score	FCN	Encoder	Encoder	ResNet	ResNet	ResNet	Encoder
F2-score	FCN	Encoder	Encoder	ResNet	ResNet	ResNet	Encoder
**65112**							
AUC	ResNet	ResNet	MLP	ResNet	MCDCNN	MCDCNN	Encoder
F1-score	ResNet	ResNet	MLP	ResNet	MCDCNN	MCDCNN	Encoder
F2-score	ResNet	Encoder	Encoder	Encoder	MCDCNN	MCDCNN	Encoder
**75350**							
AUC	MCDCNN	Encoder	Encoder	MCDCNN	ResNet	Encoder	FCN
F1-score	MCDCNN	Encoder	Encoder	Encoder	ResNet	Encoder	FCN
F2-score	T-LeNet	Encoder	TimeCNN	Encoder	MLP	Encoder	FCN
**75557**							
AUC	T-LeNet	FCN	ResNet	ResNet	T-LeNet	Encoder	T-LeNet
F1-score	MCDCNN	FCN	ResNet	ResNet	T-LeNet	Encoder	T-LeNet
F2-score	T-LeNet	FCN	ResNet	ResNet	T-LeNet	Encoder	T-LeNet
**83013**							
AUC	MLP	Encoder	Encoder	Encoder	Encoder	Encoder	Encoder
F1-score	MLP	Encoder	Encoder	Encoder	Encoder	Encoder	Encoder
F2-score	MLP	Encoder	Encoder	Encoder	Encoder	Encoder	Encoder

**Table 3:** Comparison of cross-patient average stacking performance under different input settings and final classifiers.

Input Type/Classifier	F1-Score	G-Mean
**Baseline**		
Logistic Regression	0.594	0.561
SVM	0.586	0.560
KNN	0.582	0.564
CatBoost	0.595	0.575
**SHAP+Original**		
Logistic Regression	0.598	0.564
SVM	0.604	0.594
KNN	0.605	0.576
CatBoost	0.659	0.634
**SHAP-only**		
Logistic Regression	0.785	0.728
SVM	0.654	0.531
KNN	0.642	0.535
CatBoost	0.582	0.500

## Data Availability

The data used in this study were obtained from the Medical Information Mart for Intensive Care III (MIMIC-III) Clinical Database, which is publicly available through PhysioNet. Access to the MIMIC-III database is restricted to credentialed users who have completed the required training and signed the PhysioNet Data Use Agreement.

## Supplementary Materials

The following supporting information can be downloaded at https://www.mdpi.com/article/10.3390/bioengineering13070816/s1. Table S1 reports the performance comparison under different final classifiers and input settings. Table S2 reports the effects of different SHAP source models. Table S3 reports the effects of different training data sizes used for SHAP feature generation. Table S4 compares the PCA+Original and SHAP+Original feature settings. Table S5 compares the PCA-only and SHAP-only feature settings.
