# Supplementary material for: SHAP-Based Feature Augmentation and Stacking Ensemble Learning for ECG-Based Serum Potassium Abnormality Prediction
Source: Bioengineering (Basel). 2026 Jul 16;13(7):816. doi: 10.3390/bioengineering13070816 (PMC13404188; doi:10.3390/bioengineering13070816)
Supplement: Supplementary file 1 [file bioengineering-13-00816-s001.zip › bioengineering-4375042-supplementary.pdf]

# Supplementary Material

Table S1: Performance evaluation of different final classifiers under different input types for eight patients from MIMIC-III

| Patient ID/ Classifier | Input Type | Precision | Recall | AUC | F1-score | ΔF1 | G-Mean | ΔG-Mean |
|---|---|---|---|---|---|---|---|---|
| **46092** | | | | | | | | |
| Logistic Regression | Baseline | 0.7480 | 0.6028 | 0.8339 | 0.6676 | – | 0.7336 | – |
| Logistic Regression | SHAP+Original | 0.7611 | 0.6464 | 0.8331 | 0.6991 | +0.0315 | 0.7597 | +0.0261 |
| Logistic Regression | SHAP-only | 0.7868 | 0.7472 | 0.8811 | 0.7665 | +0.0989 | 0.8169 | +0.0833 |
| SVM | Baseline | 0.7857 | 0.7459 | 0.8161 | 0.7653 | – | 0.8160 | – |
| SVM | SHAP+Original | 0.7868 | 0.7472 | 0.8684 | 0.7665 | +0.0012 | 0.8169 | +0.0009 |
| SVM | SHAP-only | 0.8380 | 0.8370 | 0.8907 | 0.8375 | +0.0722 | 0.8749 | +0.0589 |
| KNN | Baseline | 0.7845 | 0.7392 | 0.8122 | 0.7612 | – | 0.8124 | – |
| KNN | SHAP+Original | 0.7861 | 0.7452 | 0.8216 | 0.7651 | +0.0039 | 0.8157 | +0.0033 |
| KNN | SHAP-only | 0.8497 | 0.3578 | 0.8389 | 0.5036 | -0.2576 | 0.5881 | -0.2243 |
| CatBoost | Baseline | 0.7852 | 0.7424 | 0.8362 | 0.7632 | – | 0.8141 | – |
| CatBoost | SHAP+Original | 0.7868 | 0.7472 | 0.8424 | 0.7665 | +0.0033 | 0.8169 | +0.0028 |
| CatBoost | SHAP-only | 0.8391 | 0.7400 | 0.8930 | 0.7864 | +0.0232 | 0.8274 | +0.0133 |
| **60274** | | | | | | | | |
| Logistic Regression | Baseline | 0.4955 | 1.0000 | 0.5930 | 0.6626 | – | 0.7691 | – |
| Logistic Regression | SHAP+Original | 0.4955 | 1.0000 | 0.5930 | 0.6626 | 0.0000 | 0.7691 | 0.0000 |
| Logistic Regression | SHAP-only | 1.0000 | 0.9964 | 0.9993 | 0.9982 | +0.3356 | 0.9982 | +0.2291 |
| SVM | Baseline | 0.4955 | 1.0000 | 0.5930 | 0.6626 | – | 0.7691 | – |
| SVM | SHAP+Original | 0.4955 | 1.0000 | 0.5944 | 0.6626 | 0.0000 | 0.7691 | 0.0000 |
| SVM | SHAP-only | 1.0000 | 0.9964 | 0.9993 | 0.9982 | +0.3356 | 0.9982 | +0.2291 |
| KNN | Baseline | 0.4955 | 1.0000 | 0.7958 | 0.6626 | – | 0.7691 | – |
| KNN | SHAP+Original | 0.4955 | 1.0000 | 0.7958 | 0.6626 | 0.0000 | 0.7691 | 0.0000 |
| KNN | SHAP-only | 1.0000 | 0.9964 | 0.9982 | 0.9982 | +0.3356 | 0.9982 | +0.2291 |
| CatBoost | Baseline | 0.4955 | 1.0000 | 0.5937 | 0.6626 | – | 0.7691 | – |
| CatBoost | SHAP+Original | 0.5018 | 0.9964 | 0.9985 | 0.6675 | +0.0049 | 0.7753 | +0.0062 |
| CatBoost | SHAP-only | 1.0000 | 0.9964 | 0.9971 | 0.9982 | +0.3356 | 0.9982 | +0.2291 |
| **75557** | | | | | | | | |
| Logistic Regression | Baseline | 0.4136 | 0.4973 | 0.6398 | 0.4516 | – | 0.6543 | – |
| Logistic Regression | SHAP+Original | 0.4166 | 0.4964 | 0.6335 | 0.4530 | +0.0014 | 0.6544 | +0.0001 |
| Logistic Regression | SHAP-only | 0.6205 | 0.4865 | 0.7505 | 0.5454 | +0.0938 | 0.6767 | +0.0224 |
| SVM | Baseline | 0.3726 | 0.4980 | 0.6346 | 0.4263 | – | 0.6446 | – |
| SVM | SHAP+Original | 0.4162 | 0.4975 | 0.6486 | 0.4532 | +0.0269 | 0.6550 | +0.0104 |
| SVM | SHAP-only | 0.7716 | 0.4818 | 0.7290 | 0.5932 | +0.1669 | 0.6843 | +0.0397 |
| KNN | Baseline | 0.4111 | 0.4975 | 0.6729 | 0.4502 | – | 0.6538 | – |
| KNN | SHAP+Original | 0.5169 | 0.4955 | 0.6878 | 0.5060 | +0.0558 | 0.6710 | +0.0172 |
| KNN | SHAP-only | 0.7500 | 0.0007 | 0.5003 | 0.0014 | -0.4488 | 0.0260 | -0.6278 |
| CatBoost | Baseline | 0.3689 | 0.4984 | 0.6418 | 0.4240 | – | 0.6438 | – |
| CatBoost | SHAP+Original | 0.6641 | 0.4881 | 0.7193 | 0.5626 | +0.1386 | 0.6814 | +0.0376 |
| CatBoost | SHAP-only | 0.0000 | 0.0000 | 0.7447 | 0.0000 | -0.4240 | 0.0000 | -0.6438 |
| **13593** | | | | | | | | |
| Logistic Regression | Baseline | 0.2657 | 0.9861 | 0.4484 | 0.4186 | – | 0.5743 | – |
| Logistic Regression | SHAP+Original | 0.2657 | 0.9861 | 0.4654 | 0.4186 | 0.0000 | 0.5743 | 0.0000 |
| Logistic Regression | SHAP-only | 0.8847 | 0.9895 | 0.9868 | 0.9342 | +0.5156 | 0.9790 | +0.4047 |
| SVM | Baseline | 0.2520 | 0.9861 | 0.3932 | 0.4014 | – | 0.5302 | – |
| SVM | SHAP+Original | 0.2529 | 0.9861 | 0.3959 | 0.4026 | +0.0012 | 0.5334 | +0.0032 |
| SVM | SHAP-only | 0.0000 | 0.0000 | 0.9780 | 0.0000 | -0.4014 | 0.0000 | -0.5302 |
| KNN | Baseline | 0.1800 | 0.5958 | 0.4558 | 0.2765 | – | 0.4481 | – |
| KNN | SHAP+Original | 0.1813 | 0.5958 | 0.5191 | 0.2780 | +0.0015 | 0.4521 | +0.0040 |
| KNN | SHAP-only | 0.3723 | 0.6098 | 0.6793 | 0.4624 | +0.1859 | 0.6758 | +0.2277 |
| CatBoost | Baseline | 0.2228 | 0.9930 | 0.4957 | 0.3640 | – | 0.3911 | – |
| CatBoost | SHAP+Original | 0.2827 | 0.9861 | 0.8775 | 0.4394 | +0.0754 | 0.6193 | +0.2282 |
| CatBoost | SHAP-only | 0.0213 | 0.0035 | 0.9545 | 0.0060 | -0.3580 | 0.0579 | -0.3332 |

**Table S1.** *Cont.*

| Patient ID/ Classifier | Input Type | Precision | Recall | AUC | F1-score | ΔF1 | G-Mean | ΔG-Mean |
|---|---|---|---|---|---|---|---|---|
| **18996** | | | | | | | | |
| Logistic Regression | Baseline | 0.5373 | 1.0000 | 0.9973 | 0.6991 | – | 0.6956 | – |
| Logistic Regression | SHAP+Original | 0.5373 | 1.0000 | 0.9973 | 0.6991 | 0.0000 | 0.6956 | 0.0000 |
| Logistic Regression | SHAP-only | 0.5618 | 1.0000 | 0.9973 | 0.7194 | +0.0203 | 0.7297 | +0.0341 |
| SVM | Baseline | 0.4625 | 1.0000 | 0.9204 | 0.6325 | – | 0.5507 | – |
| SVM | SHAP+Original | 0.5546 | 1.0000 | 0.9194 | 0.7135 | +0.0810 | 0.7201 | +0.1694 |
| SVM | SHAP-only | 0.5618 | 1.0000 | 0.9981 | 0.7194 | +0.0869 | 0.7297 | +0.1790 |
| KNN | Baseline | 0.5453 | 1.0000 | 0.7662 | 0.7057 | – | 0.7071 | – |
| KNN | SHAP+Original | 0.5606 | 1.0000 | 0.7662 | 0.7184 | +0.0127 | 0.7281 | +0.0210 |
| KNN | SHAP-only | 0.9664 | 1.0000 | 0.9896 | 0.9829 | +0.2772 | 0.9895 | +0.2824 |
| CatBoost | Baseline | 0.5618 | 1.0000 | 0.9946 | 0.7194 | – | 0.7297 | – |
| CatBoost | SHAP+Original | 0.5606 | 1.0000 | 0.9975 | 0.7184 | -0.0010 | 0.7281 | -0.0016 |
| CatBoost | SHAP-only | 0.7135 | 1.0000 | 0.9942 | 0.8328 | +0.1134 | 0.8714 | +0.1417 |
| **65112** | | | | | | | | |
| Logistic Regression | Baseline | 0.6750 | 0.6651 | 0.8819 | 0.6700 | – | 0.7452 | – |
| Logistic Regression | SHAP+Original | 0.6750 | 0.6651 | 0.9109 | 0.6700 | 0.0000 | 0.7452 | 0.0000 |
| Logistic Regression | SHAP-only | 0.9942 | 0.9708 | 0.9755 | 0.9824 | +0.3124 | 0.9839 | +0.2387 |
| SVM | Baseline | 0.6099 | 0.6737 | 0.8709 | 0.6402 | – | 0.7239 | – |
| SVM | SHAP+Original | 0.6670 | 0.6734 | 0.8775 | 0.6702 | +0.0300 | 0.7461 | +0.0222 |
| SVM | SHAP-only | 0.8114 | 0.9734 | 0.9825 | 0.8851 | +0.2449 | 0.9273 | +0.2034 |
| KNN | Baseline | 0.6291 | 0.6714 | 0.7939 | 0.6496 | – | 0.7310 | – |
| KNN | SHAP+Original | 0.7214 | 0.7772 | 0.8009 | 0.7483 | +0.0987 | 0.8105 | +0.0795 |
| KNN | SHAP-only | 0.9997 | 0.9714 | 0.9864 | 0.9853 | +0.3357 | 0.9855 | +0.2545 |
| CatBoost | Baseline | 0.6646 | 0.6655 | 0.8714 | 0.6650 | – | 0.7418 | – |
| CatBoost | SHAP+Original | 0.9903 | 0.7768 | 0.9133 | 0.8706 | +0.2056 | 0.8796 | +0.1378 |
| CatBoost | SHAP-only | 0.9877 | 0.6375 | 0.8244 | 0.7749 | +0.1099 | 0.7968 | +0.0550 |
| **75350** | | | | | | | | |
| Logistic Regression | Baseline | 0.6716 | 0.9356 | 0.7366 | 0.7819 | – | 0.2465 | – |
| Logistic Regression | SHAP+Original | 0.6719 | 0.9417 | 0.7345 | 0.7842 | +0.0023 | 0.2380 | -0.0085 |
| Logistic Regression | SHAP-only | 0.6714 | 1.0000 | 0.8416 | 0.8034 | +0.0215 | 0.0000 | -0.2465 |
| SVM | Baseline | 0.6915 | 0.8433 | 0.7275 | 0.7599 | – | 0.4416 | – |
| SVM | SHAP+Original | 0.6919 | 0.8446 | 0.7398 | 0.7607 | +0.0008 | 0.4420 | +0.0004 |
| SVM | SHAP-only | 0.6717 | 0.9997 | 0.8223 | 0.8035 | +0.0436 | 0.0371 | -0.4045 |
| KNN | Baseline | 0.6676 | 0.8692 | 0.5441 | 0.7551 | – | 0.3168 | – |
| KNN | SHAP+Original | 0.6673 | 0.8868 | 0.5392 | 0.7616 | +0.0065 | 0.2926 | -0.0242 |
| KNN | SHAP-only | 0.6715 | 1.0000 | 0.5258 | 0.8035 | +0.0484 | 0.0185 | -0.2983 |
| CatBoost | Baseline | 0.6918 | 0.8445 | 0.7419 | 0.7606 | – | 0.4419 | – |
| CatBoost | SHAP+Original | 0.6702 | 0.9807 | 0.5691 | 0.7962 | +0.0356 | 0.1161 | -0.3258 |
| CatBoost | SHAP-only | 0.6714 | 1.0000 | 0.8399 | 0.8034 | +0.0428 | 0.0000 | -0.4419 |
| **83013** | | | | | | | | |
| Logistic Regression | Baseline | 0.2490 | 1.0000 | 0.7060 | 0.3988 | – | 0.0715 | – |
| Logistic Regression | SHAP+Original | 0.2490 | 1.0000 | 0.5983 | 0.3988 | 0.0000 | 0.0715 | 0.0000 |
| Logistic Regression | SHAP-only | 0.3598 | 1.0000 | 0.7060 | 0.5292 | +0.1304 | 0.6427 | +0.5712 |
| SVM | Baseline | 0.2481 | 1.0000 | 0.7070 | 0.3975 | – | 0.0000 | – |
| SVM | SHAP+Original | 0.2490 | 1.0000 | 0.7445 | 0.3988 | +0.0013 | 0.0715 | +0.0715 |
| SVM | SHAP-only | 0.2481 | 1.0000 | 0.5000 | 0.3975 | 0.0000 | 0.0000 | 0.0000 |
| KNN | Baseline | 0.2490 | 1.0000 | 0.5026 | 0.3988 | – | 0.0715 | – |
| KNN | SHAP+Original | 0.2490 | 1.0000 | 0.5026 | 0.3988 | 0.0000 | 0.0715 | 0.0000 |
| KNN | SHAP-only | 0.2481 | 1.0000 | 0.5000 | 0.3975 | -0.0013 | 0.0000 | -0.0715 |
| CatBoost | Baseline | 0.2490 | 1.0000 | 0.7060 | 0.3988 | – | 0.0715 | – |
| CatBoost | SHAP+Original | 0.2932 | 1.0000 | 0.7650 | 0.4534 | +0.0546 | 0.4523 | +0.3808 |
| CatBoost | SHAP-only | 0.2932 | 1.0000 | 0.7070 | 0.4534 | +0.0546 | 0.4523 | +0.3808 |

*Note:* ΔF1 and ΔG-Mean represent the absolute differences from the corresponding Baseline result for the same patient and final classifier. Positive values indicate performance improvement, whereas negative values indicate performance degradation. These values are provided to facilitate direct comparison among the different input types.

Table S2: Effects of augmented features generated from different SHAP source models on classification performance

| Patient ID/ SHAP Source | Precision | Recall | AUC | F1-score | ΔF1 | G-Mean | ΔG-Mean |
|---|---|---|---|---|---|---|---|
| **13593** | | | | | | | |
| Baseline (No SHAP) | 0.2657 | 0.9861 | 0.4484 | 0.4186 | – | 0.5743 | – |
| Logistic Regression | 0.2657 | 0.9861 | 0.4654 | 0.4186 | 0.0000 | 0.5743 | 0.0000 |
| SVM | 0.2657 | 0.9861 | 0.4484 | 0.4186 | 0.0000 | 0.5743 | 0.0000 |
| KNN | 0.2657 | 0.9861 | 0.4446 | 0.4186 | 0.0000 | 0.5743 | 0.0000 |
| CatBoost | 0.4417 | 0.9895 | 0.9875 | 0.6108 | +0.1922 | 0.8290 | +0.2547 |
| **18996** | | | | | | | |
| Baseline (No SHAP) | 0.5373 | 1.0000 | 0.9973 | 0.6991 | – | 0.6956 | – |
| Logistic Regression | 0.5373 | 1.0000 | 0.9973 | 0.6991 | 0.0000 | 0.6956 | 0.0000 |
| SVM | 0.5373 | 1.0000 | 0.9962 | 0.6991 | 0.0000 | 0.6956 | 0.0000 |
| KNN | 0.5385 | 1.0000 | 0.9977 | 0.7000 | +0.0009 | 0.6972 | +0.0016 |
| CatBoost | 0.5385 | 1.0000 | 0.9975 | 0.7000 | +0.0009 | 0.6972 | +0.0016 |
| **46092** | | | | | | | |
| Baseline (No SHAP) | 0.7480 | 0.6028 | 0.8339 | 0.6676 | – | 0.7336 | – |
| Logistic Regression | 0.7611 | 0.6464 | 0.8331 | 0.6991 | +0.0315 | 0.7597 | +0.0261 |
| SVM | 0.7487 | 0.6041 | 0.8353 | 0.6687 | +0.0011 | 0.7344 | +0.0008 |
| KNN | 0.7703 | 0.6797 | 0.8355 | 0.7221 | +0.0545 | 0.7790 | +0.0454 |
| CatBoost | 0.9948 | 0.3584 | 0.9011 | 0.5270 | -0.1406 | 0.5984 | -0.1352 |
| **60274** | | | | | | | |
| Baseline (No SHAP) | 0.4955 | 1.0000 | 0.5930 | 0.6626 | – | 0.7691 | – |
| Logistic Regression | 0.4955 | 1.0000 | 0.5930 | 0.6626 | 0.0000 | 0.7691 | 0.0000 |
| SVM | 0.4955 | 1.0000 | 0.5944 | 0.6626 | 0.0000 | 0.7691 | 0.0000 |
| KNN | 0.4955 | 1.0000 | 0.5930 | 0.6626 | 0.0000 | 0.7691 | 0.0000 |
| CatBoost | 1.0000 | 0.9964 | 0.9985 | 0.9982 | +0.3356 | 0.9982 | +0.2291 |

**Table S2.** *Cont.*

| Patient ID/ SHAP Source | Precision | Recall | AUC | F1-score | ΔF1 | G-Mean | ΔG-Mean |
|---|---|---|---|---|---|---|---|
| **65112** | | | | | | | |
| Baseline (No SHAP) | 0.6750 | 0.6651 | 0.8819 | 0.6700 | – | 0.7452 | – |
| Logistic Regression | 0.6750 | 0.6651 | 0.9109 | 0.6700 | 0.0000 | 0.7452 | 0.0000 |
| SVM | 0.6750 | 0.6651 | 0.9081 | 0.6700 | 0.0000 | 0.7452 | 0.0000 |
| KNN | 0.6750 | 0.6651 | 0.9110 | 0.6700 | 0.0000 | 0.7452 | 0.0000 |
| CatBoost | 0.9988 | 0.9709 | 0.9950 | 0.9847 | +0.3147 | 0.9851 | +0.2399 |
| **75350** | | | | | | | |
| Baseline (No SHAP) | 0.6716 | 0.9356 | 0.7366 | 0.7819 | – | 0.2465 | – |
| Logistic Regression | 0.6719 | 0.9417 | 0.7345 | 0.7842 | +0.0023 | 0.2380 | -0.0085 |
| SVM | 0.6702 | 0.9566 | 0.7473 | 0.7882 | +0.0063 | 0.1910 | -0.0555 |
| KNN | 0.6745 | 0.9536 | 0.7406 | 0.7901 | +0.0082 | 0.2381 | -0.0084 |
| CatBoost | 0.6714 | 0.9998 | 0.8484 | 0.8034 | +0.0215 | 0.0000 | -0.2465 |
| **75557** | | | | | | | |
| Baseline (No SHAP) | 0.4136 | 0.4973 | 0.6398 | 0.4516 | – | 0.6543 | – |
| Logistic Regression | 0.4166 | 0.4964 | 0.6335 | 0.4530 | +0.0014 | 0.6544 | +0.0001 |
| SVM | 0.4185 | 0.4959 | 0.6353 | 0.4539 | +0.0023 | 0.6546 | +0.0003 |
| KNN | 0.4145 | 0.4962 | 0.6415 | 0.4517 | +0.0001 | 0.6539 | -0.0004 |
| CatBoost | 0.6989 | 0.4872 | 0.7903 | 0.5741 | +0.1225 | 0.6834 | +0.0291 |
| **83013** | | | | | | | |
| Baseline (No SHAP) | 0.2490 | 1.0000 | 0.7060 | 0.3988 | – | 0.0715 | – |
| Logistic Regression | 0.2490 | 1.0000 | 0.5983 | 0.3988 | 0.0000 | 0.0715 | 0.0000 |
| SVM | 0.2490 | 1.0000 | 0.7445 | 0.3988 | 0.0000 | 0.0715 | 0.0000 |
| KNN | 0.2490 | 1.0000 | 0.7114 | 0.3988 | 0.0000 | 0.0715 | 0.0000 |
| CatBoost | 0.3559 | 1.0000 | 0.5971 | 0.5249 | +0.1261 | 0.6347 | +0.5632 |

*Note:* ΔF1 and ΔG-Mean represent the absolute differences from the corresponding Baseline (No SHAP) result for the same patient. Positive values indicate performance improvement,

whereas negative values indicate performance degradation. These values are provided to directly show the effect of using SHAP features generated from different source models.

Table S3: Effects of data size used for SHAP-augmented feature generation on classification performance

| Patient ID/ Training Size | Precision | Recall | AUC | F1-score | ΔF1 | G-Mean | ΔG-Mean |
|---|---|---|---|---|---|---|---|
| **13593** | | | | | | | |
| Train 10% | 0.1811 | 0.5958 | 0.4315 | 0.2778 | – | 0.4515 | – |
| Train 30% | 0.2657 | 0.9861 | 0.4472 | 0.4186 | +0.1408 | 0.5743 | +0.1228 |
| Train 50% | 0.2657 | 0.9861 | 0.4468 | 0.4186 | +0.1408 | 0.5743 | +0.1228 |
| Train 100% | 0.2657 | 0.9861 | 0.4654 | 0.4186 | +0.1408 | 0.5743 | +0.1228 |
| **18996** | | | | | | | |
| Train 10% | 0.5373 | 1.0000 | 0.9962 | 0.6991 | – | 0.6956 | – |
| Train 30% | 0.5373 | 1.0000 | 0.9973 | 0.6991 | 0.0000 | 0.6956 | 0.0000 |
| Train 50% | 0.5373 | 1.0000 | 0.9969 | 0.6991 | 0.0000 | 0.6956 | 0.0000 |
| Train 100% | 0.5373 | 1.0000 | 0.9973 | 0.6991 | 0.0000 | 0.6956 | 0.0000 |
| **46092** | | | | | | | |
| Train 10% | 0.6828 | 0.4348 | 0.8289 | 0.5312 | – | 0.6232 | – |
| Train 30% | 0.7465 | 0.5975 | 0.8336 | 0.6638 | +0.1326 | 0.7304 | +0.1072 |
| Train 50% | 0.7483 | 0.6028 | 0.8335 | 0.6677 | +0.1365 | 0.7336 | +0.1104 |
| Train 100% | 0.7611 | 0.6464 | 0.8331 | 0.6991 | +0.1679 | 0.7597 | +0.1365 |
| **60274** | | | | | | | |
| Train 10% | 0.4946 | 0.9964 | 0.5930 | 0.6610 | – | 0.7677 | – |
| Train 30% | 0.4955 | 1.0000 | 0.5930 | 0.6626 | +0.0016 | 0.7691 | +0.0014 |
| Train 50% | 0.4955 | 1.0000 | 0.5930 | 0.6626 | +0.0016 | 0.7691 | +0.0014 |
| Train 100% | 0.4955 | 1.0000 | 0.5930 | 0.6626 | +0.0016 | 0.7691 | +0.0014 |
| **65112** | | | | | | | |
| Train 10% | 0.6750 | 0.6651 | 0.9077 | 0.6700 | – | 0.7452 | – |
| Train 30% | 0.6750 | 0.6651 | 0.9108 | 0.6700 | 0.0000 | 0.7452 | 0.0000 |
| Train 50% | 0.6750 | 0.6651 | 0.9109 | 0.6700 | 0.0000 | 0.7452 | 0.0000 |
| Train 100% | 0.6750 | 0.6651 | 0.9109 | 0.6700 | 0.0000 | 0.7452 | 0.0000 |
| **75350** | | | | | | | |
| Train 10% | 0.6750 | 0.9588 | 0.7534 | 0.7922 | – | 0.2325 | – |
| Train 30% | 0.6800 | 0.9524 | 0.7540 | 0.7935 | +0.0013 | 0.2832 | +0.0507 |
| Train 50% | 0.6792 | 0.9533 | 0.7464 | 0.7933 | +0.0011 | 0.2763 | +0.0438 |
| Train 100% | 0.6719 | 0.9417 | 0.7345 | 0.7842 | -0.0080 | 0.2380 | +0.0055 |
| **75557** | | | | | | | |
| Train 10% | 0.4118 | 0.4962 | 0.6349 | 0.4501 | – | 0.6533 | – |
| Train 30% | 0.4110 | 0.4962 | 0.6349 | 0.4496 | -0.0005 | 0.6531 | -0.0002 |
| Train 50% | 0.4156 | 0.4962 | 0.6349 | 0.4523 | +0.0022 | 0.6541 | +0.0008 |
| Train 100% | 0.4166 | 0.4964 | 0.6335 | 0.4530 | +0.0029 | 0.6544 | +0.0011 |
| **83013** | | | | | | | |
| Train 10% | 0.2490 | 1.0000 | 0.5983 | 0.3988 | – | 0.0715 | – |
| Train 30% | 0.2490 | 1.0000 | 0.5983 | 0.3988 | 0.0000 | 0.0715 | 0.0000 |
| Train 50% | 0.2490 | 1.0000 | 0.5983 | 0.3988 | 0.0000 | 0.0715 | 0.0000 |
| Train 100% | 0.2490 | 1.0000 | 0.5983 | 0.3988 | 0.0000 | 0.0715 | 0.0000 |

*Note:* ΔF1 and ΔG-Mean represent the absolute differences from the corresponding Train 10% result for the same patient. Positive values indicate performance improvement, whereas negative values indicate performance degradation. These values are provided to show how performance changes as the amount of training data used for SHAP-augmented feature generation increases.

Table S4: Comparison of classification performance under PCA- and SHAP-based feature augmentation settings for eight patients

| Patient ID/ Classifier | Input Type | Precision | Recall | AUC | F1-score | ΔF1 | G-Mean | ΔG-Mean |
|---|---|---|---|---|---|---|---|---|
| **13593** | | | | | | | | |
| Logistic Regression | Baseline | 0.2657 | 0.9861 | 0.4484 | 0.4186 | – | 0.5743 | – |
| Logistic Regression | PCA+Original | 0.0192 | 0.0418 | 0.2952 | 0.0263 | -0.3923 | 0.1414 | -0.4329 |
| Logistic Regression | SHAP+Original | 0.2657 | 0.9861 | 0.4654 | 0.4186 | 0.0000 | 0.5743 | 0.0000 |
| SVM | Baseline | 0.2520 | 0.9861 | 0.3932 | 0.4014 | – | 0.5302 | – |
| SVM | PCA+Original | 0.0204 | 0.0418 | 0.3085 | 0.0274 | -0.3740 | 0.1459 | -0.3843 |
| SVM | SHAP+Original | 0.2529 | 0.9861 | 0.3959 | 0.4026 | +0.0012 | 0.5334 | +0.0032 |
| KNN | Baseline | 0.1800 | 0.5958 | 0.4558 | 0.2765 | – | 0.4481 | – |
| KNN | PCA+Original | 0.0192 | 0.0418 | 0.1787 | 0.0263 | -0.2502 | 0.1415 | -0.3066 |
| KNN | SHAP+Original | 0.1813 | 0.5958 | 0.5191 | 0.2780 | +0.0015 | 0.4521 | +0.0040 |
| CatBoost | Baseline | 0.2228 | 0.9930 | 0.4957 | 0.3640 | – | 0.3911 | – |
| CatBoost | PCA+Original | 0.2675 | 0.9861 | 0.3476 | 0.4208 | +0.0568 | 0.5794 | +0.1883 |
| CatBoost | SHAP+Original | 0.2827 | 0.9861 | 0.8775 | 0.4394 | +0.0754 | 0.6193 | +0.2282 |
| **18996** | | | | | | | | |
| Logistic Regression | Baseline | 0.5373 | 1.0000 | 0.9973 | 0.6991 | – | 0.6956 | – |
| Logistic Regression | PCA+Original | 0.5534 | 1.0000 | 0.9981 | 0.7125 | +0.0134 | 0.7185 | +0.0229 |
| Logistic Regression | SHAP+Original | 0.5373 | 1.0000 | 0.9973 | 0.6991 | 0.0000 | 0.6956 | 0.0000 |
| SVM | Baseline | 0.4625 | 1.0000 | 0.9204 | 0.6325 | – | 0.5507 | – |
| SVM | PCA+Original | 0.5522 | 1.0000 | 0.7705 | 0.7115 | +0.0790 | 0.7169 | +0.1662 |
| SVM | SHAP+Original | 0.5546 | 1.0000 | 0.9194 | 0.7135 | +0.0810 | 0.7201 | +0.1694 |
| KNN | Baseline | 0.5453 | 1.0000 | 0.7662 | 0.7057 | – | 0.7071 | – |
| KNN | PCA+Original | 0.5534 | 1.0000 | 0.7593 | 0.7125 | +0.0068 | 0.7185 | +0.0114 |
| KNN | SHAP+Original | 0.5606 | 1.0000 | 0.7662 | 0.7184 | +0.0127 | 0.7281 | +0.0210 |
| CatBoost | Baseline | 0.5618 | 1.0000 | 0.9946 | 0.7194 | – | 0.7297 | – |
| CatBoost | PCA+Original | 0.5558 | 1.0000 | 0.9981 | 0.7145 | -0.0049 | 0.7217 | -0.0080 |
| CatBoost | SHAP+Original | 0.5606 | 1.0000 | 0.9975 | 0.7184 | -0.0010 | 0.7281 | -0.0016 |
| **75350** | | | | | | | | |
| Logistic Regression | Baseline | 0.6716 | 0.9356 | 0.7366 | 0.7819 | – | 0.2465 | – |
| Logistic Regression | PCA+Original | 0.7809 | 0.9087 | 0.8129 | 0.8400 | +0.0581 | 0.6598 | +0.4133 |
| Logistic Regression | SHAP+Original | 0.6719 | 0.9417 | 0.7345 | 0.7842 | +0.0023 | 0.2380 | -0.0085 |
| SVM | Baseline | 0.6915 | 0.8433 | 0.7275 | 0.7599 | – | 0.4416 | – |
| SVM | PCA+Original | 0.7708 | 0.9124 | 0.8010 | 0.8357 | +0.0758 | 0.6377 | +0.1961 |
| SVM | SHAP+Original | 0.6919 | 0.8446 | 0.7398 | 0.7607 | +0.0008 | 0.4420 | +0.0004 |
| KNN | Baseline | 0.6676 | 0.8692 | 0.5441 | 0.7551 | – | 0.3168 | – |
| KNN | PCA+Original | 0.7703 | 0.9181 | 0.6973 | 0.8377 | +0.0826 | 0.6360 | +0.3192 |
| KNN | SHAP+Original | 0.6673 | 0.8868 | 0.5392 | 0.7616 | +0.0065 | 0.2926 | -0.0242 |
| CatBoost | Baseline | 0.6918 | 0.8445 | 0.7419 | 0.7606 | – | 0.4419 | – |
| CatBoost | PCA+Original | 0.7437 | 0.9166 | 0.7713 | 0.8211 | +0.0605 | 0.5699 | +0.1280 |
| CatBoost | SHAP+Original | 0.6702 | 0.9807 | 0.5691 | 0.7962 | +0.0356 | 0.1161 | -0.3258 |
| **46092** | | | | | | | | |
| Logistic Regression | Baseline | 0.7480 | 0.6028 | 0.8339 | 0.6676 | – | 0.7336 | – |
| Logistic Regression | PCA+Original | 0.7852 | 0.7463 | 0.8487 | 0.7652 | +0.0976 | 0.8160 | +0.0824 |
| Logistic Regression | SHAP+Original | 0.7611 | 0.6464 | 0.8331 | 0.6991 | +0.0315 | 0.7597 | +0.0261 |
| SVM | Baseline | 0.7857 | 0.7459 | 0.8161 | 0.7653 | – | 0.8160 | – |
| SVM | PCA+Original | 0.7864 | 0.7476 | 0.8500 | 0.7665 | +0.0012 | 0.8170 | +0.0010 |
| SVM | SHAP+Original | 0.7868 | 0.7472 | 0.8684 | 0.7665 | +0.0012 | 0.8169 | +0.0009 |
| KNN | Baseline | 0.7845 | 0.7392 | 0.8122 | 0.7612 | – | 0.8124 | – |
| KNN | PCA+Original | 0.7856 | 0.7482 | 0.8184 | 0.7664 | +0.0052 | 0.8170 | +0.0046 |
| KNN | SHAP+Original | 0.7861 | 0.7452 | 0.8216 | 0.7651 | +0.0039 | 0.8157 | +0.0033 |
| CatBoost | Baseline | 0.7852 | 0.7424 | 0.8362 | 0.7632 | – | 0.8141 | – |
| CatBoost | PCA+Original | 0.7864 | 0.7476 | 0.8375 | 0.7665 | +0.0033 | 0.8170 | +0.0029 |
| CatBoost | SHAP+Original | 0.7868 | 0.7472 | 0.8424 | 0.7665 | +0.0033 | 0.8169 | +0.0028 |

Table S4: *Cont.*

| Patient ID/ Classifier | Input Type | Precision | Recall | AUC | F1-score | ΔF1 | G-Mean | ΔG-Mean |
|---|---|---|---|---|---|---|---|---|
| **60274** | | | | | | | | |
| Logistic Regression | Baseline | 0.4955 | 1.0000 | 0.5930 | 0.6626 | – | 0.7691 | – |
| Logistic Regression | PCA+Original | 0.4918 | 0.9854 | 0.5937 | 0.6561 | -0.0065 | 0.7635 | -0.0056 |
| Logistic Regression | SHAP+Original | 0.4955 | 1.0000 | 0.5930 | 0.6626 | 0.0000 | 0.7691 | 0.0000 |
| SVM | Baseline | 0.4955 | 1.0000 | 0.5930 | 0.6626 | – | 0.7691 | – |
| SVM | PCA+Original | 0.4946 | 0.9964 | 0.5937 | 0.6610 | -0.0016 | 0.7677 | -0.0014 |
| SVM | SHAP+Original | 0.4955 | 1.0000 | 0.5944 | 0.6626 | 0.0000 | 0.7691 | 0.0000 |
| KNN | Baseline | 0.4955 | 1.0000 | 0.7958 | 0.6626 | – | 0.7691 | – |
| KNN | PCA+Original | 0.4918 | 0.9854 | 0.7917 | 0.6561 | -0.0065 | 0.7635 | -0.0056 |
| KNN | SHAP+Original | 0.4955 | 1.0000 | 0.7958 | 0.6626 | 0.0000 | 0.7691 | 0.0000 |
| CatBoost | Baseline | 0.4955 | 1.0000 | 0.5937 | 0.6626 | – | 0.7691 | – |
| CatBoost | PCA+Original | 0.4946 | 0.9964 | 0.5937 | 0.6610 | -0.0016 | 0.7677 | -0.0014 |
| CatBoost | SHAP+Original | 0.5018 | 0.9964 | 0.9985 | 0.6675 | +0.0049 | 0.7753 | +0.0062 |
| **65112** | | | | | | | | |
| Logistic Regression | Baseline | 0.6750 | 0.6651 | 0.8819 | 0.6700 | – | 0.7452 | – |
| Logistic Regression | PCA+Original | 0.6352 | 0.7716 | 0.8851 | 0.6968 | +0.0268 | 0.7715 | +0.0263 |
| Logistic Regression | SHAP+Original | 0.6750 | 0.6651 | 0.9109 | 0.6700 | 0.0000 | 0.7452 | 0.0000 |
| SVM | Baseline | 0.6099 | 0.6737 | 0.8709 | 0.6402 | – | 0.7239 | – |
| SVM | PCA+Original | 0.6346 | 0.7713 | 0.9040 | 0.6963 | +0.0561 | 0.7711 | +0.0472 |
| SVM | SHAP+Original | 0.6670 | 0.6734 | 0.8775 | 0.6702 | +0.0300 | 0.7461 | +0.0222 |
| KNN | Baseline | 0.6291 | 0.6714 | 0.7939 | 0.6496 | – | 0.7310 | – |
| KNN | PCA+Original | 0.6619 | 0.7728 | 0.8036 | 0.7130 | +0.0634 | 0.7845 | +0.0535 |
| KNN | SHAP+Original | 0.7214 | 0.7772 | 0.8009 | 0.7483 | +0.0987 | 0.8105 | +0.0795 |
| CatBoost | Baseline | 0.6646 | 0.6655 | 0.8714 | 0.6650 | – | 0.7418 | – |
| CatBoost | PCA+Original | 0.7083 | 0.8197 | 0.8354 | 0.7600 | +0.0950 | 0.8228 | +0.0810 |
| CatBoost | SHAP+Original | 0.9903 | 0.7768 | 0.9133 | 0.8706 | +0.2056 | 0.8796 | +0.1378 |
| **75557** | | | | | | | | |
| Logistic Regression | Baseline | 0.4136 | 0.4973 | 0.6398 | 0.4516 | – | 0.6543 | – |
| Logistic Regression | PCA+Original | 0.5412 | 0.4908 | 0.6369 | 0.5148 | +0.0632 | 0.6712 | +0.0169 |
| Logistic Regression | SHAP+Original | 0.4166 | 0.4964 | 0.6335 | 0.4530 | +0.0014 | 0.6544 | +0.0001 |
| SVM | Baseline | 0.3726 | 0.4980 | 0.6346 | 0.4263 | – | 0.6446 | – |
| SVM | PCA+Original | 0.5388 | 0.4941 | 0.7034 | 0.5155 | +0.0892 | 0.6730 | +0.0284 |
| SVM | SHAP+Original | 0.4162 | 0.4975 | 0.6486 | 0.4532 | +0.0269 | 0.6550 | +0.0104 |
| KNN | Baseline | 0.4111 | 0.4975 | 0.6729 | 0.4502 | – | 0.6538 | – |
| KNN | PCA+Original | 0.5395 | 0.4932 | 0.7053 | 0.5154 | +0.0652 | 0.6725 | +0.0187 |
| KNN | SHAP+Original | 0.5169 | 0.4955 | 0.6878 | 0.5060 | +0.0558 | 0.6710 | +0.0172 |
| CatBoost | Baseline | 0.3689 | 0.4984 | 0.6418 | 0.4240 | – | 0.6438 | – |
| CatBoost | PCA+Original | 0.5359 | 0.4941 | 0.6498 | 0.5142 | +0.0902 | 0.6726 | +0.0288 |
| CatBoost | SHAP+Original | 0.6641 | 0.4881 | 0.7193 | 0.5626 | +0.1386 | 0.6814 | +0.0376 |
| **83013** | | | | | | | | |
| Logistic Regression | Baseline | 0.2490 | 1.0000 | 0.7060 | 0.3988 | – | 0.0715 | – |
| Logistic Regression | PCA+Original | 0.3274 | 1.0000 | 0.7006 | 0.4933 | +0.0945 | 0.5677 | +0.4962 |
| Logistic Regression | SHAP+Original | 0.2490 | 1.0000 | 0.5983 | 0.3988 | 0.0000 | 0.0715 | 0.0000 |
| SVM | Baseline | 0.2481 | 1.0000 | 0.7070 | 0.3975 | – | 0.0000 | – |
| SVM | PCA+Original | 0.3258 | 1.0000 | 0.7057 | 0.4933 | +0.0958 | 0.5677 | +0.5677 |
| SVM | SHAP+Original | 0.2490 | 1.0000 | 0.7445 | 0.3988 | +0.0013 | 0.0715 | +0.0715 |
| KNN | Baseline | 0.2490 | 1.0000 | 0.5026 | 0.3988 | – | 0.0715 | – |
| KNN | PCA+Original | 0.3274 | 1.0000 | 0.6611 | 0.4933 | +0.0945 | 0.5677 | +0.4962 |
| KNN | SHAP+Original | 0.2490 | 1.0000 | 0.5026 | 0.3988 | 0.0000 | 0.0715 | 0.0000 |
| CatBoost | Baseline | 0.2490 | 1.0000 | 0.7060 | 0.3988 | – | 0.0715 | – |
| CatBoost | PCA+Original | 0.3287 | 1.0000 | 0.7060 | 0.4947 | +0.0959 | 0.5710 | +0.4995 |
| CatBoost | SHAP+Original | 0.2932 | 1.0000 | 0.7650 | 0.4534 | +0.0546 | 0.4523 | +0.3808 |

*Note:* ΔF1 and ΔG-Mean represent the absolute differences from the corresponding Baseline result for the same patient and final classifier. Positive values indicate performance improvement, whereas negative values indicate performance degradation. These values are provided to facilitate direct comparison between the PCA+Original and SHAP+Original feature augmentation settings.

Table S5: Comparison of classification performance under PCA-only and SHAP-only feature settings for eight patients

| Patient ID/ Classifier | Input Type | Precision | Recall | AUC | F1-score | ΔF1 | G-Mean | ΔG-Mean |
|---|---|---|---|---|---|---|---|---|
| **13593** | | | | | | | | |
| Logistic Regression | Baseline | 0.2657 | 0.9861 | 0.4484 | 0.4186 | – | 0.5743 | – |
| Logistic Regression | PCA-only | 0.0207 | 0.0418 | 0.2943 | 0.0276 | -0.3910 | 0.1468 | -0.4275 |
| Logistic Regression | SHAP-only | 0.8847 | 0.9895 | 0.9868 | 0.9342 | +0.5156 | 0.9790 | +0.4047 |
| SVM | Baseline | 0.2520 | 0.9861 | 0.3932 | 0.4014 | – | 0.5302 | – |
| SVM | PCA-only | 0.0112 | 0.0244 | 0.3072 | 0.0154 | -0.3860 | 0.1076 | -0.4226 |
| SVM | SHAP-only | 0.0000 | 0.0000 | 0.9780 | 0.0000 | -0.4014 | 0.0000 | -0.5302 |
| KNN | Baseline | 0.1800 | 0.5958 | 0.4558 | 0.2765 | – | 0.4481 | – |
| KNN | PCA-only | 0.0145 | 0.0314 | 0.2548 | 0.0198 | -0.2567 | 0.1225 | -0.3256 |
| KNN | SHAP-only | 0.3723 | 0.6098 | 0.6793 | 0.4624 | +0.1859 | 0.6758 | +0.2277 |
| CatBoost | Baseline | 0.2228 | 0.9930 | 0.4957 | 0.3640 | – | 0.3911 | – |
| CatBoost | PCA-only | 0.0033 | 0.0070 | 0.2248 | 0.0045 | -0.3595 | 0.0582 | -0.3329 |
| CatBoost | SHAP-only | 0.0213 | 0.0035 | 0.9545 | 0.0060 | -0.3580 | 0.0579 | -0.3332 |
| **18996** | | | | | | | | |
| Logistic Regression | Baseline | 0.5373 | 1.0000 | 0.9973 | 0.6991 | – | 0.6956 | – |
| Logistic Regression | PCA-only | 0.5534 | 1.0000 | 0.9981 | 0.7125 | +0.0134 | 0.7185 | +0.0229 |
| Logistic Regression | SHAP-only | 0.5618 | 1.0000 | 0.9973 | 0.7194 | +0.0203 | 0.7297 | +0.0341 |
| SVM | Baseline | 0.4625 | 1.0000 | 0.9204 | 0.6325 | – | 0.5507 | – |
| SVM | PCA-only | 0.5511 | 1.0000 | 0.7336 | 0.7106 | +0.0781 | 0.7152 | +0.1645 |
| SVM | SHAP-only | 0.5618 | 1.0000 | 0.9981 | 0.7194 | +0.0869 | 0.7297 | +0.1790 |
| KNN | Baseline | 0.5453 | 1.0000 | 0.7662 | 0.7057 | – | 0.7071 | – |
| KNN | PCA-only | 0.5522 | 1.0000 | 0.7581 | 0.7115 | +0.0058 | 0.7169 | +0.0098 |
| KNN | SHAP-only | 0.9664 | 1.0000 | 0.9896 | 0.9829 | +0.2772 | 0.9895 | +0.2824 |
| CatBoost | Baseline | 0.5618 | 1.0000 | 0.9946 | 0.7194 | – | 0.7297 | – |
| CatBoost | PCA-only | 0.5522 | 1.0000 | 0.9981 | 0.7115 | -0.0079 | 0.7169 | -0.0128 |
| CatBoost | SHAP-only | 0.7135 | 1.0000 | 0.9942 | 0.8328 | +0.1134 | 0.8714 | +0.1417 |
| **75350** | | | | | | | | |
| Logistic Regression | Baseline | 0.6716 | 0.9356 | 0.7366 | 0.7819 | – | 0.2465 | – |
| Logistic Regression | PCA-only | 0.7821 | 0.9206 | 0.8253 | 0.8458 | +0.0639 | 0.6619 | +0.4154 |
| Logistic Regression | SHAP-only | 0.6714 | 1.0000 | 0.8416 | 0.8034 | +0.0215 | 0.0000 | -0.2465 |
| SVM | Baseline | 0.6915 | 0.8433 | 0.7275 | 0.7599 | – | 0.4416 | – |
| SVM | PCA-only | 0.8057 | 0.8144 | 0.7094 | 0.8100 | +0.0501 | 0.6982 | +0.2566 |
| SVM | SHAP-only | 0.6717 | 0.9997 | 0.8223 | 0.8035 | +0.0436 | 0.0371 | -0.4045 |
| KNN | Baseline | 0.6676 | 0.8692 | 0.5441 | 0.7551 | – | 0.3168 | – |
| KNN | PCA-only | 0.7865 | 0.8983 | 0.6112 | 0.8387 | +0.0836 | 0.6713 | +0.3545 |
| KNN | SHAP-only | 0.6715 | 1.0000 | 0.5258 | 0.8035 | +0.0484 | 0.0185 | -0.2983 |
| CatBoost | Baseline | 0.6918 | 0.8445 | 0.7419 | 0.7606 | – | 0.4419 | – |
| CatBoost | PCA-only | 0.7923 | 0.8902 | 0.7873 | 0.8384 | +0.0778 | 0.6823 | +0.2404 |
| CatBoost | SHAP-only | 0.6714 | 1.0000 | 0.8399 | 0.8034 | +0.0428 | 0.0000 | -0.4419 |
| **46092** | | | | | | | | |
| Logistic Regression | Baseline | 0.7480 | 0.6028 | 0.8339 | 0.6676 | – | 0.7336 | – |
| Logistic Regression | PCA-only | 0.7439 | 0.6500 | 0.7607 | 0.6938 | +0.0262 | 0.7571 | +0.0235 |
| Logistic Regression | SHAP-only | 0.7868 | 0.7472 | 0.8811 | 0.7665 | +0.0989 | 0.8169 | +0.0833 |
| SVM | Baseline | 0.7857 | 0.7459 | 0.8161 | 0.7653 | – | 0.8160 | – |
| SVM | PCA-only | 0.7660 | 0.6668 | 0.8308 | 0.7130 | -0.0523 | 0.7714 | -0.0446 |
| SVM | SHAP-only | 0.8380 | 0.8370 | 0.8907 | 0.8375 | +0.0722 | 0.8749 | +0.0589 |
| KNN | Baseline | 0.7845 | 0.7392 | 0.8122 | 0.7612 | – | 0.8124 | – |
| KNN | PCA-only | 0.7537 | 0.7219 | 0.8106 | 0.7374 | -0.0238 | 0.7949 | -0.0175 |
| KNN | SHAP-only | 0.8497 | 0.3578 | 0.8389 | 0.5036 | -0.2576 | 0.5881 | -0.2243 |
| CatBoost | Baseline | 0.7852 | 0.7424 | 0.8362 | 0.7632 | – | 0.8141 | – |
| CatBoost | PCA-only | 0.7490 | 0.6716 | 0.8341 | 0.7082 | -0.0550 | 0.7693 | -0.0448 |
| CatBoost | SHAP-only | 0.8391 | 0.7400 | 0.8930 | 0.7864 | +0.0232 | 0.8274 | +0.0133 |

Table S5: *Cont.*

| Patient ID/ Classifier | Input Type | Precision | Recall | AUC | F1-score | ΔF1 | G-Mean | ΔG-Mean |
|---|---|---|---|---|---|---|---|---|
| **60274** | | | | | | | | |
| Logistic Regression | Baseline | 0.4955 | 1.0000 | 0.5930 | 0.6626 | – | 0.7691 | – |
| Logistic Regression | PCA-only | 0.4918 | 0.9854 | 0.5937 | 0.6561 | -0.0065 | 0.7635 | -0.0056 |
| Logistic Regression | SHAP-only | 1.0000 | 0.9964 | 0.9993 | 0.9982 | +0.3356 | 0.9982 | +0.2291 |
| SVM | Baseline | 0.4955 | 1.0000 | 0.5930 | 0.6626 | – | 0.7691 | – |
| SVM | PCA-only | 0.4946 | 0.9964 | 0.9933 | 0.6610 | -0.0016 | 0.7677 | -0.0014 |
| SVM | SHAP-only | 1.0000 | 0.9964 | 0.9993 | 0.9982 | +0.3356 | 0.9982 | +0.2291 |
| KNN | Baseline | 0.4955 | 1.0000 | 0.7958 | 0.6626 | – | 0.7691 | – |
| KNN | PCA-only | 0.4918 | 0.9854 | 0.7917 | 0.6561 | -0.0065 | 0.7635 | -0.0056 |
| KNN | SHAP-only | 1.0000 | 0.9964 | 0.9982 | 0.9982 | +0.3356 | 0.9982 | +0.2291 |
| CatBoost | Baseline | 0.4955 | 1.0000 | 0.5937 | 0.6626 | – | 0.7691 | – |
| CatBoost | PCA-only | 0.4946 | 0.9964 | 0.7939 | 0.6610 | -0.0016 | 0.7677 | -0.0014 |
| CatBoost | SHAP-only | 1.0000 | 0.9964 | 0.9971 | 0.9982 | +0.3356 | 0.9982 | +0.2291 |
| **65112** | | | | | | | | |
| Logistic Regression | Baseline | 0.6750 | 0.6651 | 0.8819 | 0.6700 | – | 0.7452 | – |
| Logistic Regression | PCA-only | 0.7060 | 0.7690 | 0.8847 | 0.7362 | +0.0662 | 0.8013 | +0.0561 |
| Logistic Regression | SHAP-only | 0.9942 | 0.9708 | 0.9755 | 0.9824 | +0.3124 | 0.9839 | +0.2387 |
| SVM | Baseline | 0.6099 | 0.6737 | 0.8709 | 0.6402 | – | 0.7239 | – |
| SVM | PCA-only | 0.6347 | 0.7715 | 0.8938 | 0.6964 | +0.0562 | 0.7712 | +0.0473 |
| SVM | SHAP-only | 0.8114 | 0.9734 | 0.9825 | 0.8851 | +0.2449 | 0.9273 | +0.2034 |
| KNN | Baseline | 0.6291 | 0.6714 | 0.7939 | 0.6496 | – | 0.7310 | – |
| KNN | PCA-only | 0.6001 | 0.7703 | 0.8349 | 0.6746 | +0.0250 | 0.7526 | +0.0216 |
| KNN | SHAP-only | 0.9997 | 0.9714 | 0.9864 | 0.9853 | +0.3357 | 0.9855 | +0.2545 |
| CatBoost | Baseline | 0.6646 | 0.6655 | 0.8714 | 0.6650 | – | 0.7418 | – |
| CatBoost | PCA-only | 0.7146 | 0.7115 | 0.8499 | 0.7131 | +0.0481 | 0.7793 | +0.0375 |
| CatBoost | SHAP-only | 0.9877 | 0.6375 | 0.8244 | 0.7749 | +0.1099 | 0.7968 | +0.0550 |
| **75557** | | | | | | | | |
| Logistic Regression | Baseline | 0.4136 | 0.4973 | 0.6398 | 0.4516 | – | 0.6543 | – |
| Logistic Regression | PCA-only | 0.5497 | 0.4908 | 0.6391 | 0.5186 | +0.0670 | 0.6722 | +0.0179 |
| Logistic Regression | SHAP-only | 0.6205 | 0.4865 | 0.7505 | 0.5454 | +0.0938 | 0.6767 | +0.0224 |
| SVM | Baseline | 0.3726 | 0.4980 | 0.6346 | 0.4263 | – | 0.6446 | – |
| SVM | PCA-only | 0.5686 | 0.4894 | 0.7833 | 0.5260 | +0.0997 | 0.6734 | +0.0288 |
| SVM | SHAP-only | 0.7716 | 0.4818 | 0.7290 | 0.5932 | +0.1669 | 0.6843 | +0.0397 |
| KNN | Baseline | 0.4111 | 0.4975 | 0.6729 | 0.4502 | – | 0.6538 | – |
| KNN | PCA-only | 0.5495 | 0.4910 | 0.7080 | 0.5186 | +0.0684 | 0.6723 | +0.0185 |
| KNN | SHAP-only | 0.7500 | 0.0007 | 0.5003 | 0.0014 | -0.4488 | 0.0260 | -0.6278 |
| CatBoost | Baseline | 0.3689 | 0.4984 | 0.6418 | 0.4240 | – | 0.6438 | – |
| CatBoost | PCA-only | 0.5682 | 0.4908 | 0.6356 | 0.5266 | +0.1026 | 0.6743 | +0.0305 |
| CatBoost | SHAP-only | 0.0000 | 0.0000 | 0.7447 | 0.0000 | -0.4240 | 0.0000 | -0.6438 |
| **83013** | | | | | | | | |
| Logistic Regression | Baseline | 0.2490 | 1.0000 | 0.7060 | 0.3988 | – | 0.0715 | – |
| Logistic Regression | PCA-only | 0.3564 | 1.0000 | 0.6994 | 0.5255 | +0.1267 | 0.6357 | +0.5642 |
| Logistic Regression | SHAP-only | 0.3598 | 1.0000 | 0.7060 | 0.5292 | +0.1304 | 0.6427 | +0.5712 |
| SVM | Baseline | 0.2481 | 1.0000 | 0.7070 | 0.3975 | – | 0.0000 | – |
| SVM | PCA-only | 0.3564 | 1.0000 | 0.7060 | 0.5255 | +0.1280 | 0.6357 | +0.6357 |
| SVM | SHAP-only | 0.2481 | 1.0000 | 0.5000 | 0.3975 | 0.0000 | 0.0000 | 0.0000 |
| KNN | Baseline | 0.2490 | 1.0000 | 0.5026 | 0.3988 | – | 0.0715 | – |
| KNN | PCA-only | 0.3564 | 1.0000 | 0.7020 | 0.5255 | +0.1267 | 0.6357 | +0.5642 |
| KNN | SHAP-only | 0.2481 | 1.0000 | 0.5000 | 0.3975 | -0.0013 | 0.0000 | -0.0715 |
| CatBoost | Baseline | 0.2490 | 1.0000 | 0.7060 | 0.3988 | – | 0.0715 | – |
| CatBoost | PCA-only | 0.3287 | 1.0000 | 0.6611 | 0.4947 | +0.0959 | 0.5710 | +0.4995 |
| CatBoost | SHAP-only | 0.2932 | 1.0000 | 0.7070 | 0.4534 | +0.0546 | 0.4523 | +0.3808 |

*Note:* ΔF1 and ΔG-Mean represent the absolute differences from the corresponding Baseline result for the same patient and final classifier. Positive values indicate performance improvement, whereas negative values indicate performance degradation. These values are provided to facilitate direct comparison between the PCA-only and SHAP-only feature settings.
